# Reconstitution of actomyosin networks in cell-sized liposomes reveals distinct mechanical roles of cytoskeletal organization in membrane shape remodeling

**DOI:** 10.1101/2025.05.18.654456

**Published:** 2025-05-22

**Authors:** Makito Miyazaki, Fahmida Sultana Laboni, Taeyoon Kim

**Affiliations:** 1 RIKEN Center for Integrative Medical Sciences, 1-7-22 Suehiro-cho, Tsurumi-ku, Yokohama, Kanagawa 230-0045, Japan.; 2 RIKEN Center for Biosystems Dynamics Research, 2-2-3 Minatojima-minamimachi, Chuo-ku, Kobe, Hyogo 650-0047, Japan.; 3 Graduate School of Medicine, Science and Technology, Shinshu University, 3-1-1 Asahi, Matsumoto, Nagano 390-8621, Japan.; 4 PRESTO, JST, 4-1-8 Honcho, Kawaguchi, Saitama 332-0012, Japan.; 5 Weldon School of Biomedical Engineering, Purdue University, West Lafayette, Indiana 47907-2032, USA.; 6 EMBRIO Institute, Purdue University, West Lafayette, Indiana 47907-2032, USA.; 7 Faculty of Science and Technology, Keio University, Kohoku-ku, Yokohama, Kanagawa 223-0061, Japan.

## Abstract

The actin cortex, a thin layer of actomyosin network beneath the plasma membrane, regulates various cell functions by generating active forces and inducing membrane deformations, including blebs. Although upstream signaling is involved in regulating cell shape, the extent to which downstream actomyosin molecules can control the shape remains elusive. Here, using a minimal reconstituted system with a combination of agent-based computational model, we show that while actin-membrane coupling strength determines the magnitude of membrane deformation, its balance with actin network connectivity governs the bleb initiation mechanism, either by detachment of the cortex from the membrane or by rupture of the cortex. This balance also regulates whether single or multiple blebs form. Furthermore, both experiments and simulations suggest that not only the dense cortical network but also the sparse volume-spanning network actively contributes to regulating bleb number. These findings provide mechanical insights into how cells tune actin network organization to control their shape.

## INTRODUCTION

Cells assemble diverse structures of actin networks to operate biological functions. Among these structures, one of the most ubiquitous structures is the actin cortex, a thin layer of an actomyosin network formed beneath the plasma membrane^1^. The actin cortex is not only a passive scaffold that stabilizes the cell shape, but also plays pivotal roles in regulating various biological functions, such as migration, division, and polarity establishment, by generating active contractile forces ^1,2,3,4^. These forces drive morphological transitions of cells, including the formation of spherical membrane protrusions called blebs. During apoptosis, cells form transient multiple blebs in random directions^5^. By contrast, in many other processes, the number and position of blebs are tightly regulated. During cell migration mediated by the actin cortex, primordial germ cells create a single bleb with a size comparable to the cell body at the front end to drive migration^6,7,8^. Similarly, during amoeboid migration under physical confinement, various epithelial and mesenchymal cells such as HeLa cells also form a single bleb^9^. In contrast, Walker 293 carcinosarcoma cells^10,11^ and *Dictyostelium* cells^12,13^ create relatively small multiple blebs at the leading edge. During cell division, cells form multiple blebs near the poles, possibly to relieve intracellular pressure elevated by furrow ingression and ensure successful cytokinesis^14^.

How can the same key molecular elements of the actin cortex, namely actin filaments (F-actins) and myosin motors, induce various morphological transitions with tunable properties? This remains an open question. Although the upstream biochemical signaling pathways mediated by Rho-family GTPases and phosphoinositides are involved in the regulations^3,5,15^, downstream proteins in the actin cortex directly mediate the morphological transition. Thus, it is important to ascertain the extent to which physical aspects of the actin cortex can control the cell shape. One of the promising but challenging strategies is a bottom-up approach: reconstituting the process from a minimal set of proteins and cell-sized liposomes^16,17^. It has been demonstrated that liposomes containing actin and myosin exhibit different patterns of contraction depending on the actin-membrane interaction strength^18^, and that the addition of anillin induces detectable membrane deformations including blebs^19^. However, these studies primarily focused on post-contraction steady-states and lacked quantitative time-lapse analysis. Recent studies reported bleb formation upon laser ablation of the cortical actin network^20^ or myosin activation by light-induced degradation of a myosin inhibitor^21^. However, these systems could not reconstitute spontaneous bleb formation. As an alternative approach, agent-based computational models have been employed. To recapitulate bleb formation, cells were often simplified as structures consisting of a membrane, actin cortex, and cytoplasm with internal cytoskeleton^23,24,25^. However, these models could not reproduce large bleb formation because it did not account for membrane fluidity, which permits movement of anchor points between the membrane and the cortex. Although some models have incorporated membrane fluidity^26,27^, the cytoskeletal networks were drastically simplified, limiting the capability and predictability.

Here, we investigate the physical mechanism of morphological transition driven by the actin cortex, combining a minimal reconstituted model with an agent-based computational model. Our reconstituted system, consisting of purified cytoskeletal proteins and cell-sized liposomes, enables independent control of i) actin-membrane coupling strength, ii) actin network connectivity, and iii) actin network distribution inside liposomes. Unlike previous reconstituted systems, myosin activities are temporally regulated by myosin kinase, mimicking regulation in living cells. The agent-based computational model, consisting of an actomyosin network and a deformable membrane, enables molecular-scale spatiotemporal analysis, providing mechanical insights inaccessible to experiments. Using these advantages, we systematically investigate how each parameter affects the morphological transition process.

## RESULTS

### Reconstituting the actin cortex inside a cell-sized liposome.

**Figure 1.**
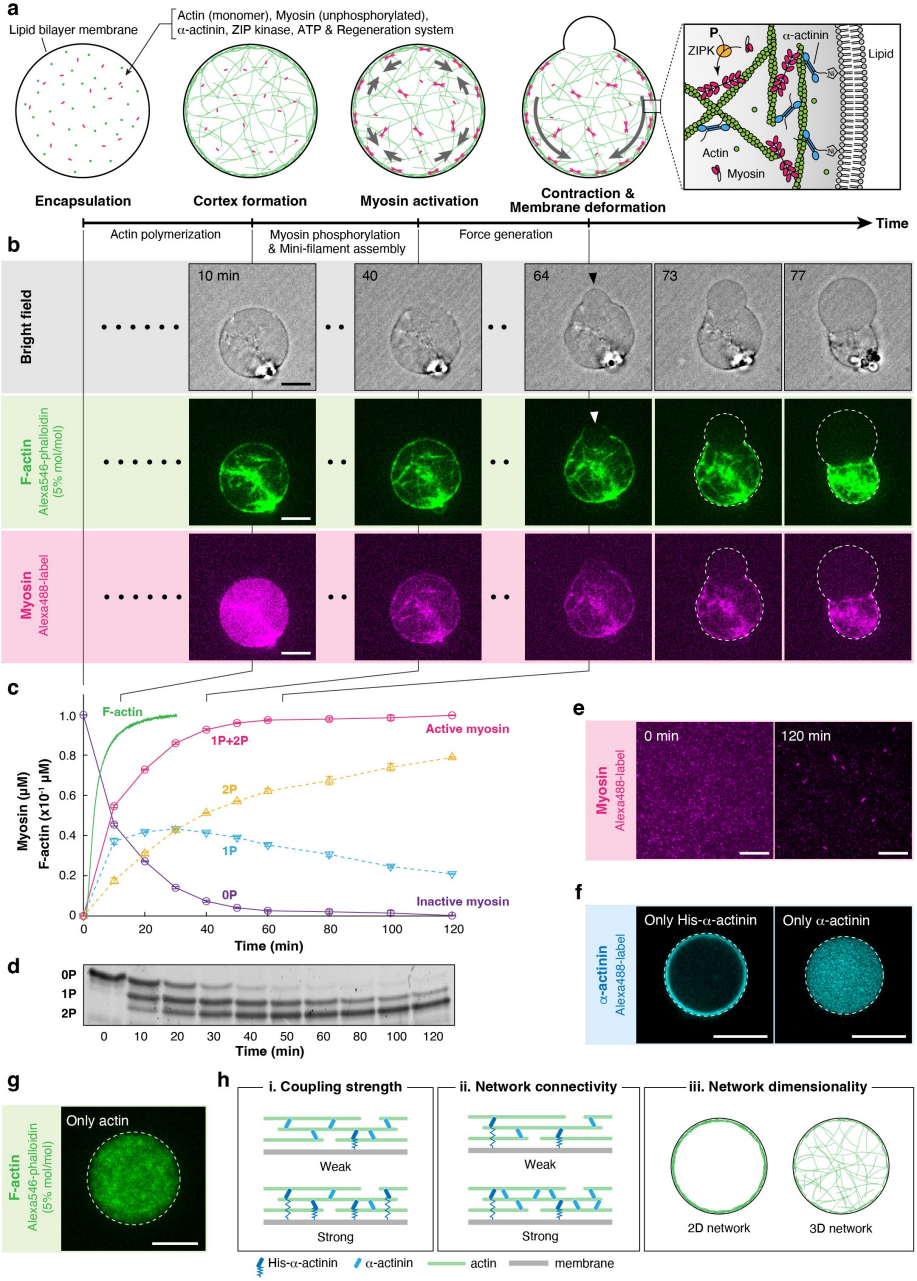
Reconstitution of the active actin cortex inside a cell-sized liposome. **a,** Schematic illustration of the experimental system. Actin monomers were mixed on ice with myosin, α-actinin (unphosphorylated), ZIP kinase, ATP, and ATP regeneration system. The mixture was immediately encapsulated into a cell-sized liposome by the inverted emulsion method, and the temperature was elevated to 25 ± 1°C to promote actin polymerization and activate myosin contractility. The membrane was composed of 85% phosphatidylcholine (PC), 10% phosphatidylglycerol (PG), and 5% Ni-NTA-conjugated lipid. **b,** Time-lapse confocal images of a liposome at the equator showing actin cortex formation, followed by myosin localization on F-actin, cortex contraction, and bleb formation and expansion (**Movie S1**). A black arrowhead and a white arrowhead indicate the bleb and the rupture point of the actin cortex, respectively. The liposome contains 10 μM actin, 2 μM His-α-actinin, 1 μM SMM (10% Alexa488-labeled), and 1.4 × 10^−4^ unit ml^−1^ ZIPK. **c,** Biochemical assays showing the kinetics of actin polymerization and myosin phosphorylation. Actin polymerization was monitored using pyrene-labeled actin. Myosin phosphorylation was quantified from gel electrophoresis shown in **d**. Three independent experiments were performed. Error bars indicate SDs. **d,** The image of urea/glycerol PAGE. 1 μM myosin was incubated with 1.4 × 10^−4^ unit ml^−1^ ZIPK in the presence of 1 mM ATP for the indicated times at 25°C. Unphosphorylated (0P), monophosphorylated (1P), and diphosphorylated (2P) myosin regulatory light chains were separated by gel electrophoresis. **e,** Total internal reflection fluorescence (TIRF) images of myosin before (left) and after (right) 120 min incubation with 1.4 × 10^−4^ unit ml^−1^ ZIPK in the presence of 1 mM ATP and ATP regeneration system. Submicrometer-long mini-filaments were formed by phosphorylation (Fig. S1b). **f,** Confocal images of liposomes at the equator containing only (left) his-tagged or (right) his-tag truncated α-actinin, showing that α-actinin was anchored to the membrane through the his-tag and Ni-NTA interaction, as illustrated in the inset of **a**. **g,** Confocal images of a liposome at the equator containing only actin, showing that F-actin has no specific interaction with the membrane. **h,** Schematic illustration of three physical parameters examined in the experiments. For all microscopic images, dashed lines indicate the liposome periphery. Scale bars, 10 μm.

We developed a minimal model of the actin cortex by reconstituting a contractile actomyosin network from purified proteins (Fig. S1a) inside a cell-sized liposome (Fig. 1a). In cells, myosin contractility is regulated by phosphorylation of the myosin regulatory light chain by myosin kinases^28^. Phosphorylated myosin dimers assemble into submicrometer-long filaments that generate contractile forces on F-actin^28^ (Fig. 1a, inset). To recapitulate this regulation, we used ZIP kinase^29^ (ZIPK), one of the major myosin kinases involved in cell motility^30^, cytokinesis^31,32^, and apoptosis^33^. We tuned the ZIPK concentration to let the cortex formation and contraction processes take place sequentially (Fig. 1b, **Movie S1**). We confirmed by biochemical assays^34,35^ (Fig. 1c,d) that 90% of 10 μM actin was polymerized ~10 min after the encapsulation (Fig. 1c, green line), whereas it took ~40 min to phosphorylate 90% of 1 μM myosin (Fig. 1c, magenta line). We also confirmed by fluorescence microscopy that myosin self-assembled into submicrometer-long filaments upon its phosphorylation (Fig. 1e, Fig. S1b).

F-actin in the cortex is inter-connected by various crosslinking proteins, including α-actinin, fimbrin, plastin, and fascin. The filaments are also anchored to the plasma membrane by diverse actin-membrane linkers such as ezrin-radixin-moesin (ERM) proteins and anillin^1^. Among the crosslinking proteins, α-actinin is the most abundant actin crosslinker in the actin cortex, as revealed by the proteomic analysis^36,37^, and is involved in diverse morphological transition processes, including cell motility^38,39^, division^40^, and mitotic cell rounding^41^. We therefore used α-actinin to create a crosslinked network within a liposome. α-Actinin also binds to the plasma membrane through its pleckstrin-homology (PH), which has affinity for phosphatidylinositol 4,5-bisphosphate (PI(4,5)P_2_)^42,43^. To control this interaction, we instead used a defined linkage between a histidine-tag introduced to the recombinant α-actinin and Ni-NTA-conjugated phospholipids in the membrane (Fig. 1a, inset). We confirmed that, while his-tagged α-actinin (hereafter “His-α-actinin”) binds to the membrane (Fig. 1f, left), his-tag-truncated α-actinin (hereafter “α-actinin”) does not bind to the membrane (Fig. 1f, right). We also confirmed that F-actin shows no specific interactions with the membrane (Fig. 1g).

In subsequent experiments, we fixed the concentrations of actin ($C_{A}$) and myosin ($C_{M}$), and ZIPK at 10 μM, 1 μM, and 1.4 × 10^−4^ unit ml^−1^, respectively. Then, by varying the concentrations of His-α-actinin ($C_{H}$) and α-actinin ($C_{N}$), we independently controlled actin-membrane coupling strength, defined as $R_{C}=C_{H}/C_{A}$ (Fig. 1h, i) and actin network connectivity, defined as $R_{X}=C_{X}/C_{A}$ (Fig. 1h, ii), where $C_{X}=C_{H}+C_{N}$ is the total crosslinker concentration. A co-sedimentation assay showed that the apparent dissociation constant $K_{d}$ between His-α-actinin and F-actin is 1.4 μM, with binding to 10 μM actin reaching saturation at ~5 μM (Fig. S1c,d). Therefore, we set the maximum $C_{X}$ at 5 μM ($R_{X}=0.5$). Furthermore, the spatial distribution of F-actin was modulated by adding methylcellulose to the inner solution of the liposomes. This crowding agent induces the localization of F-actin beneath the membrane by the depletion effect^44,45^, thereby enabling the formation of both a two-dimensional (2D) cortical network and a three-dimensional (3D) volume-spanning network (Fig. 1h, iii).

Taking all these advantages, we systematically investigated the effects of (i) actin-membrane coupling strength, (ii) actin network connectivity, and (iii) actin network dimensionality on the morphological transition process of cell-sized liposomes. The size of observed liposomes was 19.9 ± 7.56 μm (mean ± S.D.; n=355) in diameter, comparable to the typical cell size.

### His-α-actinin concentration determines the morphological transition modes of liposomes.

**Figure 2.**
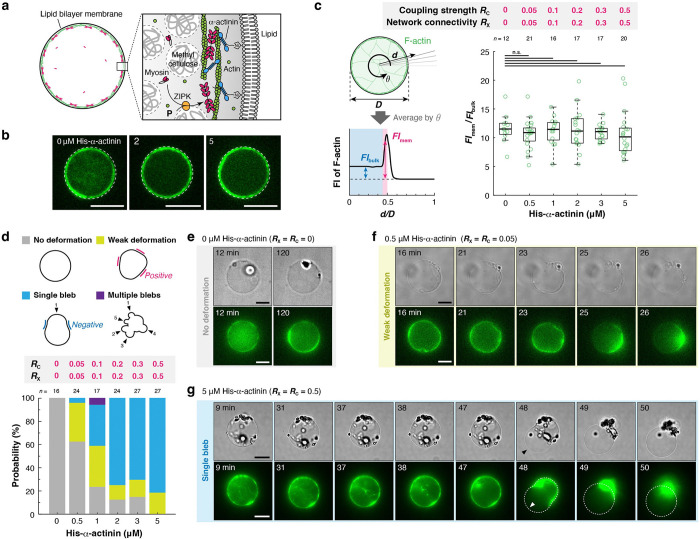
His-α-actinin concentration switches the morphological transition modes. **a,** Schematic illustration of the experimental system. Methylcellulose was added to localize F-actin beneath the membrane. **b,** Representative confocal images of liposomes at the equator showing F-actin distribution in the presence of various concentrations of His-α-actinin. **c,** Quantitative analysis of F-actin distribution. The fluorescence intensity of F-actin beneath the membrane $FI_{\text{mem}}$ divided by the fluorescence intensity of F-actin in the bulk $FI_{\text{bulk}}$ was plotted for each liposome. n.s.: *p* ≥ 0.05 (Welch’s *t*-test, two-sided). **d,** Probability distribution of the morphological transition modes of liposomes. Liposomes were classified into four categories in terms of their shapes: “No deformation”, “Weak deformation”, “Single bleb”, and “Multiple blebs”. Liposomes exhibiting negative membrane curvature at more than one location during the observation period were considered to have undergone bleb formation. These were further divided into “Single bleb” or “Multiple blebs” based on the number of blebs. Liposomes that showed detectable deformation without regions of negative curvature were classified as “Weak deformation”, while those showing no detectable deformation during the period of observation (2 hours) were assigned to “No deformation”. **e-g,** Time-lapse epifluorescence images of representative liposomes with various concentrations of His-α-actinin. **e,** No deformation (**Movie S2**), **f,** Weak deformation (**Movie S3**), and **g,** Single bleb (**Movie S4**). A black arrowhead and a white arrowhead indicate the bleb and the rupture point of the actin cortex, respectively. For all microscopic images, dashed lines indicate the liposome periphery. Scale bars, 10 μm.

We initially focused on a 2D cortical network created with methylcellulose (Fig. 2a). Only His-α-actinin was used for simplicity (i.e., $R_{X}=R_{C}$). First, we confirmed without myosin and ZIPK that His-α-actinin was co-localized with F-actin (Fig. S2a), and the actin density in a 2D cortical network remained nearly constant across the entire range of $C_{H}$ (Fig. 2b,c, Fig. S2b). Then, we performed time-lapse microscopy in the presence of myosin and ZIPK to examine how varying $C_{H}$ affects the morphological transition of liposomes (Fig. 2d–g). In the absence of His-α-actinin ($C_{H}=0$; $R_{X}=R_{C}=0$), neither global contraction of cortical actin networks nor detectable deformation of liposomes was observed (Fig. 2e, **Movie S2**). This result is consistent with previous reports, as myosin filaments are unable to induce global contraction of actin networks without actin crosslinking proteins at physiological concentration of ATP^46,47,48^ (maintained at 1 mM in our experiments). At $C_{H}=0.5\;\mu M$ ($R_{X}=R_{C}=0.05$), the cortical actin network showed contraction in 58% (14/24) of the liposomes, consistent with the lower limit α-actinin density ($R_{X}=0.05$) required for the global contraction in millimeter-scale actomyosin gels^48^. More than half of these liposomes (64%) (9/14) showed detectable deformation during contraction (Fig. 2f, **Movie S3**). A further increase in $C_{H}$ increased the fraction of liposomes showing detectable deformation from 38% ($C_{H}=0.5\;\mu M$; $R_{X}=R_{C}=0.05$) to 100% ($C_{H}=5\;\mu M$; $R_{X}=R_{C}=0.5$) (Fig. 2g, **Movie S4**).

To elucidate a relationship between $C_{H}$ and the shape of liposomes, we classified the shape into four categories: “No deformation”, “Weak deformation”, “Single bleb”, and “Multiple blebs” (Fig. 2d, top). Quantitative analysis revealed that increasing $C_{H}$ shifted the dominant mode from “No deformation” to “Weak deformation” to “Single bleb” (Fig. 2d, bottom). At the saturated $C_{H}$ (5 μM; $R_{X}=R_{C}=0.5$), all liposomes showed deformation, and 81% of liposomes (22/27) formed “Single bleb” (Fig. 2d, bottom). Formation of “Multiple blebs” was a very rare event; it was observed in only one case (1/17) at $C_{H}=1\;\mu M$ ($R_{X}=R_{C}=0.1$) (Fig. S3a, **Movie S5**).

### Actin-membrane coupling strength is a primary determinant controlling the morphological transition mode and the magnitude of membrane deformation.

**Figure 3.**
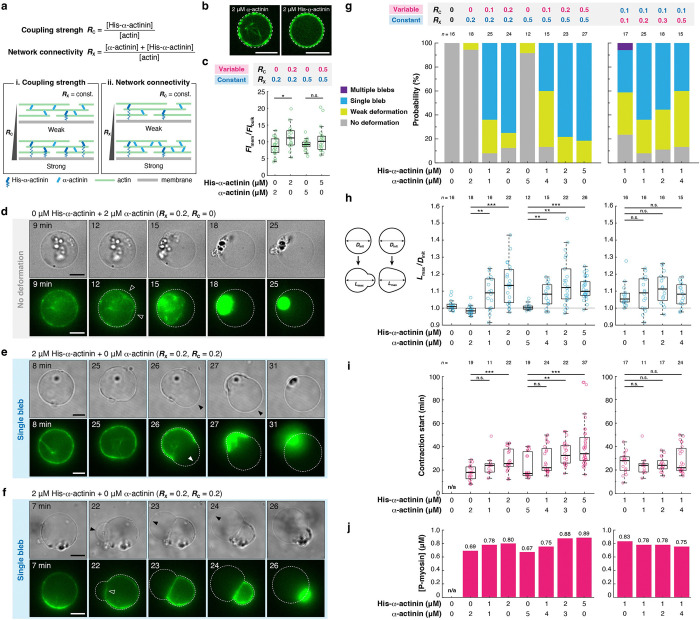
Actin-membrane coupling strength is the key parameter controlling the morphological transition mode and the magnitude of membrane deformation. **a,** Schematic illustration of the method to independently control actin-membrane coupling strength, $R_{C}$, and network connectivity, $R_{X}$. **b,** Representative confocal images of liposomes at the equator showing F-actin distribution in the presence of (left) α-actinin or (right) His-α-actinin. **c,** Quantitative analysis of F-actin distribution. The fluorescence intensity of F-actin beneath the membrane $FI_{\text{mem}}$ divided by the fluorescence intensity of F-actin in the bulk $FI_{\text{bulk}}$ was plotted for each liposome. **d-f,** Time-lapse epifluorescence images of representative liposomes under various conditions. **d,** The actin cortex was detached from the membrane (white open arrowheads). No clear membrane deformation was observed during the contraction (**Movie S6**). **e,** Rupture of the actin cortex (white filled arrowhead), followed by the contraction and blebbing of the membrane (black filled arrowheads) (**Movie S7**). **f,** Detachment of the actin cortex from the membrane (open white arrowhead), followed by the contraction and blebbing of the membrane (black filled arrowheads) (**Movie S8**). The cortex was not ruptured until the completion of contraction. **g-j,** Effects of $R_{C}$ and $R_{X}$ on (**g**) the morphological transition mode, (**h**) the magnitude of membrane deformation, (**i**) the contraction start time, and (**j**) the critical concentration of myosin required for the contraction, estimated from the median contraction start time in **i** and the time course of myosin phosphorylation (Fig. 1c). **g,** Liposomes were classified into four categories in terms of their shapes, as shown in Fig. 2d. **h,** The magnitude of membrane deformation is defined as the maximum length of the major axis of the liposome during contraction, $L_{max}$, divided by the diameter of the liposome before contraction, $D_{\text{init}}$, as illustrated on the left. For all microscopic images, dashed lines indicate the liposome periphery. Scale bars, 10 μm. ***: *p* < 0.001, **: *p* < 0.01, n.s.: *p* ≥ 0.05 (Welch’s *t*-test, two-sided).

We demonstrated that the single parameter, $C_{H}$, switched the morphological transition mode of liposomes (Fig. 2). However, changing $C_{H}$ modulates both actin-membrane coupling strength ($R_{C}$) and actin network connectivity ($R_{X}$). Therefore, it remains unknown how these two physical parameters contribute to the morphological transition mode and magnitude of membrane deformation. To dissect the role of each parameter, we mixed His-α-actinin and α-actinin at various concentrations to control these two parameters independently (Fig. 3a). Then, we quantified the morphology and magnitude of membrane deformation in various conditions. We confirmed that the density of F-actin beneath the membrane was similar regardless of $C_{H}$ and $C_{N}$ (Fig. 3b,c, Fig. S2b,c). Therefore, this experimental setup enabled us to independently control the actin-membrane coupling strength and the network connectivity without a significant change in F-actin distribution.

Using this strategy, we first examined the effects of a variation in $R_{C}$ while keeping $R_{X}$ fixed (Fig.3a, i). In the absence of actin-membrane coupling ($R_{C}=0$), most liposomes showed no detectable deformation, despite noticeable contraction of the cortical actin network at both $R_{X}=0.2$ and 0.5 (Fig. 3d, Fig. 3g, left, **Movie S6**). In the presence of actin-membrane coupling ($R_{C}>0$), liposomes were deformed by network contraction (Fig. 3e,f, Fig. 3g, left, Fig. S3b, **Movie S7, Movie S8, Movie S9**). With an increase in $R_{C}$, the dominant morphological transition mode shifted from “No deformation” to “Weak deformation”, and then to “Single bleb” at both $R_{X}=0.2$ and 0.5, which is consistent with the experiment using only His-α-actinin (Fig. 2d). At both $R_{X}=0.2$ and 0.5, the magnitude of membrane deformation increased with higher $R_{C}$ and reached a plateau at high $R_{C}$-values (Fig. 3h, left). The onset time of network contraction was delayed with increasing $R_{C}$ with both $R_{X}=0.2$ and 0.5 (Fig. 3i, left), showing a trend similar to the magnitude of deformation (Fig. 3h, left). Using the time course of myosin phosphorylation (Fig. 1c,d), we estimated the concentration of phosphorylated myosin at the onset time of contraction (Fig. 3j, left). The result showed that higher myosin activity was required to initiate membrane deformation with increased actin-membrane coupling strength. Reducing the ZIPK concentration significantly delayed the onset time of contraction (Fig.ES4a, **Movie S10**), but the estimated concentration of phosphorylated myosin at the onset time and the magnitude of membrane deformation were comparable between the two concentrations, further validating the estimation method (Fig. S4b–f).

We next examined the effects of a variation in $R_{X}$ while keeping $R_{C}$ fixed (Fig.3a, ii). We found that, in contrast to a change in $R_{C}$, changing $R_{X}$ did not show a clear shift of the morphological transition modes (Fig. 3g, right). The magnitude of membrane deformation was not affected either by $R_{X}$ (Fig. 3h, right). Notably, the onset time of network contraction was similar, regardless of $R_{X}$ (Fig. 3i, right), implying that critical myosin activity required for the membrane deformation was not strongly affected by the network connectivity in this parameter range (Fig. 3j, right). Taken together, these results demonstrate that actin-membrane coupling strength, rather than network connectivity, is the primary determinant of both the morphological transition mode and the magnitude of membrane deformation.

### Bleb formation is initiated by one of two distinct mechanisms, “Detachment” or “Rupture”, depending on the balance between actin-membrane coupling strength and network connectivity.

**Figure 4.**
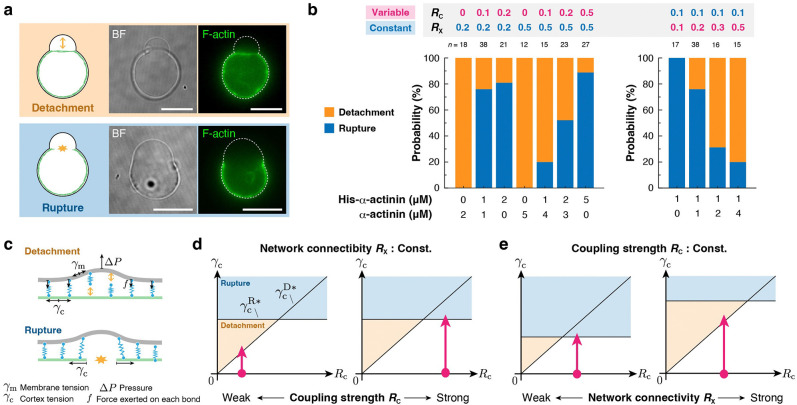
Bleb formation is induced by one of two distinct mechanisms, which is determined by the balance between actin-membrane coupling strength and network connectivity. **a,** Two types of bleb initiation mechanisms observed in the experiments. Top: A bleb is induced by the detachment of the cortical actin network from the membrane (**Movie S8, Movie S9**). Bottom: A bleb is induced by the rupture of the cortical actin network (**Movie S7**). Dashed lines indicate the liposome periphery. Scale bars, 10 μm. **b,** Probability distribution of the two types of bleb initiation mechanisms under various conditions. **c,** Schematic illustration of the molecular mechanisms underlying the two bleb initiation mechanisms. **d,e,** Phase diagrams of the bleb initiation mechanisms. $\gamma_{c}^{D*}$ and $\gamma_{c}^{R*}$ indicate the critical cortex tensions required for the detachment and rupture mechanisms, respectively. $\gamma_{c}^{D*}$ is proportional to the actin-membrane coupling strength $R_{C}$, as shown in Eq. 8. By contrast, $\gamma_{c}^{R*}$ is an increasing function of the network connectivity $R_{X}$, but it is independent of $R_{C}$. **d,** Dependence on actin-membrane coupling strength, $R_{C}$. **e,** Dependence on network connectivity, $R_{X}$. Magenta arrows indicate the pathways taken by the system upon myosin phosphorylation.

Time-lapse observation revealed that bleb formation was initiated by two distinct mechanisms: “Detachment” or “Rupture” (Fig. 4a). As for the “Rupture,” bleb formation was initiated by the spontaneous rupture of the actin cortex beneath the membrane (Fig. 3e, filled arrowhead, **Movie S7**). By contrast, as for the “Detachment,” bleb formation was initiated by the local detachment of the actin cortex from the membrane (Fig. 3f, open arrowhead, **Movie S8**). Note that, in some cases showing “Detachment,” the cortex was eventually ruptured during the bleb expansion (Fig. S3b, filled arrowhead, **Movie S9**). We found that, at constant $R_{X}$, “Detachment” was dominant at low $R_{C}$, whereas “Rupture” became dominant with increasing $R_{C}$ (Fig. 4b, left). On the contrary, at constant $R_{C}$, “Rupture” was dominant at low $R_{X}$, whereas “Rupture” became dominant with increasing $R_{X}$ (Fig. 4b, right). These results suggest that the bleb initiation mechanism is determined by how well filaments are connected in the network and how strongly the network is coupled to the membrane.

To gain physical insight into the bleb initiation mechanism, we developed a simple theoretical model. First, we introduce a theoretical description of the “Detachment” mechanism (Fig. 4c, top). We assume that a hydrostatic pressure difference between the inside and outside of the liposome, ΔP, is balanced by the elastic restoring force, F, generated by the coupling between the actin cortex and the membrane-bound His-α-actinin molecules until the time point just before bleb initiation:

(1) ΔP+F=0.

Using the Young-Laplace equation, ΔP can be written as

(2) $$\Delta P=\frac{2\left(\gamma_{m}\;+\;\gamma_{c}\right)}{R}-\frac{2\gamma_{m0}}{R_{0}},$$

where $\gamma_{m}$, $\gamma_{c}$, and R indicate the membrane tension, the actin cortex tension, and radius of the liposome at a certain time point t, respectively. $\gamma_{mo}$ and $R_{0}$ indicate the membrane tension and the radius of the liposome right after the liposome formation (t=0), respectively^14,20^. As myosin phosphorylation proceeds and the internal pressure reaches a level close to the bleb initiation threshold, the contribution of $\gamma_{m}$ would become much smaller than that of $\gamma_{c}\;\left(\gamma_{c}\gg \gamma_{m}\right)$, which was confirmed by *in silico* simulation (see Fig. 6f, Fig. 7c,g,k). Thus, the internal pressure can be approximated as

(3) $$\Delta P≃\frac{2\gamma_{c}}{R}.$$

Next, we consider the binding and unbinding dynamics of actin-membrane coupling. Although there are two potential molecular pathways for decoupling – either unbinding of His-α-actinin from F-actin or unbinding of the His-tag from Ni-NTA-lipid – we assume that the former is the predominant pathway for simplicity (see Supplementary Note 2, Section 7). In the absence of the hydrostatic pressure (ΔP=0), the actin-bound His-α-actinin density $\rho_{H}^{b}$ is governed by the kinetic equation:

(4) $$\frac{d\rho_{H}^{b}}{\;dt}=k_{on}\left(\rho_{H}-\rho_{H}^{b}\right)\left(\rho_{A}-\rho_{H}^{b}-\rho_{N}^{b}\right)-k_{off}\rho_{H}^{b},$$

where $\rho_{H}$, $\rho_{H}^{b}$, $\rho_{N}^{b}$, and $\rho_{A}$ indicate the surface densities of His-α-actinin, His-α-actinin bound on F-actin, α-actinin bound on F-actin, and F-actin beneath the membrane, respectively. $k_{on}$ and $k_{off}$ are the rate constants for binding and unbinding between His-α-actinin and F-actin, respectively. Considering that ΔP tends to separate the membrane from the actin cortex, and assuming that each His-α-actinin-F-actin bond is equally stressed, the force per bond, f, becomes $f=\Delta P/\rho_{H}^{b}$. Using Bell’s kinetics model^49^, the kinetic equation (Eq. 4) is replaced by

(5) $$\frac{d\rho_{H}^{b}}{\;dt}=k_{on}\left(\rho_{H}-\rho_{H}^{b}\right)\left(\rho_{A}-\rho_{H}^{b}-\rho_{N}^{b}\right)-k_{off}^{0}\rho_{H}^{b}exp\left(\frac{r_{0}}{k_{B}T}\frac{\Delta P}{\rho_{H}^{b}}\right),$$

where $k_{\text{off}}^{0}$ is the zero-force unbinding rate constant, and $r_{0}$ is a characteristic distance to overcome the energetic barrier for bond separation^49,50,51^. When a small constant ΔP is applied, the unbinding rate (second term on the right-hand side of Eq. 5) transiently increases, leading a slight decrease in $\rho_{H}^{b}$, and then the system reaches a new equilibrium ($d\rho_{H}^{b}/dt=0$). In contrast, when a large ΔP is applied, the unbinding rate (second term) becomes much larger than the binding rate (first term), causing $\rho_{H}^{b}$ to rapidly approach zero. There will thus exist the critical pressure, $\Delta P^{*}$, that is just sufficient to detach all His-α-actinin molecules from F-actin within a local region (Supplementary Note 1). If it is assumed that F-actin near the membrane retains many free binding sites for α-actinin $\left(\rho_{A}\gg \rho_{H}^{b}+\rho_{N}^{b}\right)$, we obtain the critical pressure:

(6) $$\Delta P^{*}≃\frac{0.7k_{B}T\rho_{H}}{r_{0}}\ln\left(\frac{\rho_{A}}{K_{m}^{2D}}\right),$$

where $K_{m}^{2D}=k_{\text{off}}^{0}/k_{\text{on}}$ is the dissociation constant of His-α-actinin from F-actin in a 2D network (Supplementary Note 1). Therefore, using Eq. 3, the critical cortical tension required for the “Detachment” mechanism is estimated as

(7) $$\gamma_{C}^{D*}≃\frac{0.35\;k_{B}T\;\rho_{H}R}{r_{0}}\ln\left(\frac{\rho_{A}}{K_{m}^{2D}}\right).$$

Since $R_{C}≃\rho_{H}/\rho_{A}$, this tension is proportional to the actin-membrane coupling strength $R_{C}$:

(8) $$\gamma_{C}^{D*}\propto R_{C}.$$

We next consider the “Rupture” mechanism (Fig. 4c, bottom). It can be reasonably approximated that the critical cortical tension required for the actin network rupture $\gamma_{C}^{R*}$ is an increasing function of $R_{X}$ and independent of $R_{C}$ in our parameter range^52^.

Based on the analytical results, we generated phase diagrams predicting which bleb initiation mechanism is selected (Fig. 4d,e). At constant $R_{X}$, the model predicts that the mechanism shifts from “Detachment” to “Rupture” with increasing $R_{C}$ (Fig. 4d). At constant $R_{C}$, the model predicts that the mechanism shifts from “Rupture” to “Detachment” with increasing $R_{X}$ (Fig. 4e). These predictions are entirely consistent with the experimental results (Fig. 4b). Furthermore, the model predicts that at constant $R_{X}$, higher myosin activity is required to initiate bleb formation at higher $R_{C}$. This is also consistent with the experimental results (Fig. 3i, left, Fig. 3j, left). Collectively, this simple theoretical model explains that the mechanism triggering the bleb initiation is regulated by the balance between actin-membrane coupling strength and network connectivity.

### 3D volume-spanning actin network facilitates the formation of multiple blebs.

**Figure 5.**
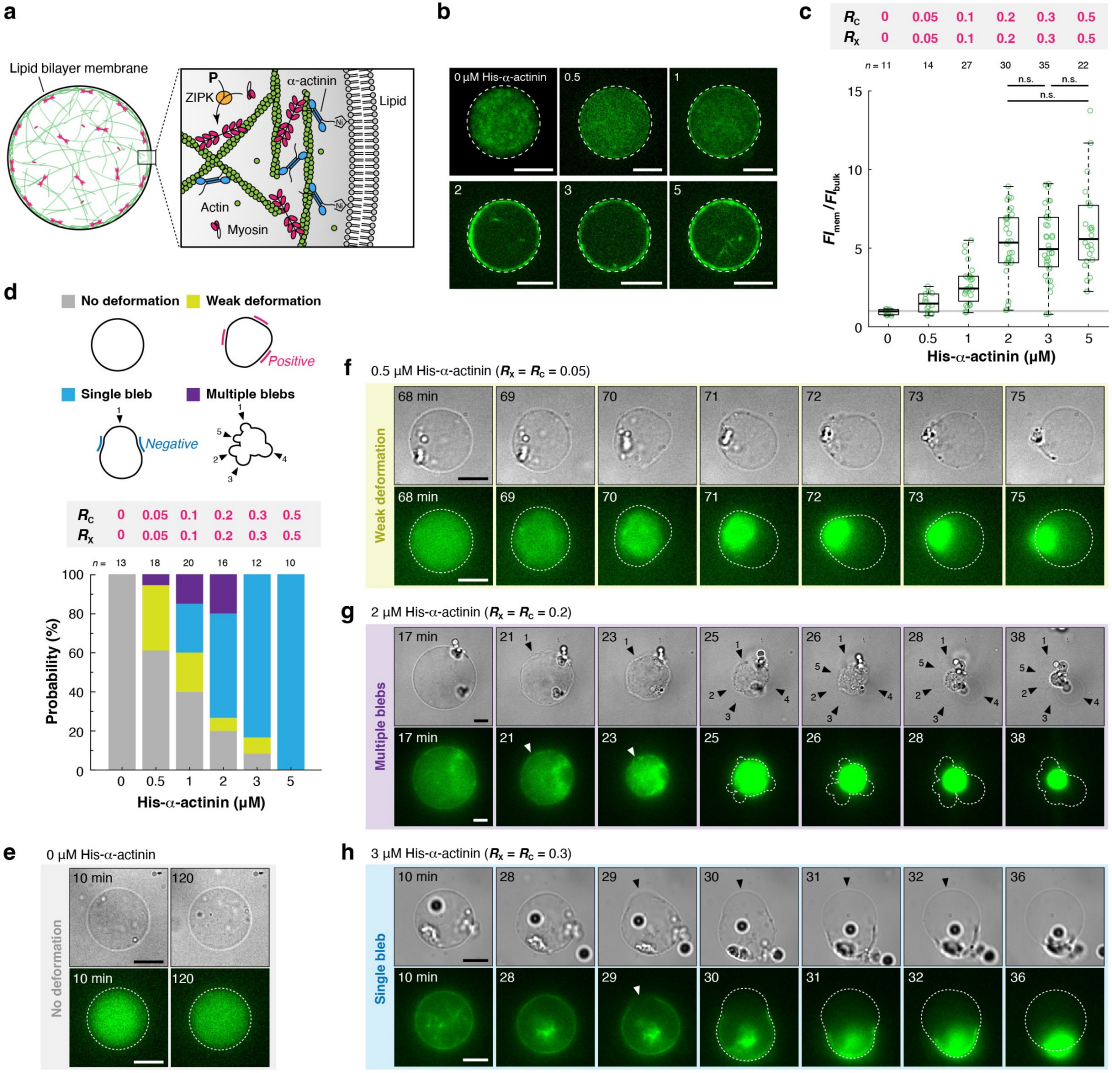
3D volume-spanning actin network facilitates the formation of multiple blebs. **a,** Schematic illustration of the experimental system. Methylcellulose was excluded from the inner solution to create a 3D volume-spanning actin network. **b,** Representative confocal images of liposomes at the equator showing F-actin distribution in the presence of various concentrations of His-α-actinin. **c,** Quantitative analysis of F-actin distribution. The fluorescence intensity of F-actin beneath the membrane $FI_{\text{mem}}$ divided by the fluorescence intensity of F-actin in the bulk $FI_{\text{bulk}}$ was plotted for each liposome. n.s.: *p* ≥ 0.05 (Welch’s *t*-test, two-sided). **d,** Probability distribution of the morphological transition modes of liposomes. Liposomes were classified into four categories in terms of their shapes, as in the cases with a 2D cortical network (Fig. 2d, Fig. 3g). **e-h,** Time-lapse epifluorescence images of representative liposomes under various concentrations of His-α-actinin. **e,** No deformation (**Movie S11**), **f,** Weak deformation (**Movie S12**), **g,** Multiple blebs (**Movie S13**), and **h,** Single bleb (**Movie S14**). Black arrowheads and white arrowheads indicate the blebs and the rupture points of the actin cortex, respectively. For all microscopic images, dashed lines indicate the liposome periphery. Scale bars, 10 μm.

In cells, F-actin not only forms a dense cortex beneath the plasma membrane but also forms a sparse volume-spanning network throughout the cytoplasm. Therefore, it is important to assess how this coexisting bulk network influences membrane deformation. To address this, we created a volume-spanning 3D network by excluding methylcellulose from the inner solution (Fig. 5a) and examined how the concentration of His-α-actinin ($C_{H}$) affects morphological transitions, as analyzed in the 2D network (Fig. 2). As in the 2D network case, only His-α-actinin was used for simplicity, (i.e., $R_{X}=R_{C}$). We first confirmed that an increase in $C_{H}$ increased the cortical F-actin density, reaching a plateau at $C_{H}=2\;\mu M$ ($R_{X}=R_{C}=0.2$) (Fig. 5b,c, Fig. S2d). However, the relative cortical F-actin density $FI_{\text{mem}}/{FI}_{\text{bulk}}$ was lower than that in the cases with methylcellulose (Fig.2c, Fig. 3c, Fig. S2e), suggesting that F-actin formed a sparse network in the bulk region even at high $C_{H}$. We next performed time-lapse microscopy to examine how $C_{H}$ affects the morphological transition of liposomes (Fig. 5d–h). In the absence of His-α-actinin ($C_{H}=0\;\mu M$; $R_{X}=R_{C}=0$), neither contraction of actin networks nor deformation of liposomes was observed (Fig. 5e, **Movie S11**). At $C_{H}=0.5\;\mu M$ ($R_{X}=R_{C}=0.05$), the actin network showed contraction in 39% (7/18) of the liposomes, and some of these liposomes deformed during the contraction (Fig. 5f, **Movie S12**). At $C_{H}=1\;\mu M$ ($R_{X}=R_{C}=0.1$), the actin network showed contraction in all the liposomes (20/20). A further increase in $C_{H}$ induced larger deformation (Fig. 5g,h **Movie S13, Movie S14**).

Similar to the 2D network (Fig. 2d), an increase in $C_{H}$ shifted the dominant mode from “No deformation” to “Weak deformation” to “Single bleb” (Fig. 5d). Indeed, at the saturated $C_{H}$ (5 μM; $R_{X}=R_{C}=0.5$), all liposomes (10/10) formed single blebs (Fig. 5h, **Movie S14**). Remarkably, at intermediate $C_{H}$ (0.5, 1, and 2 μM; $R_{X}=R_{C}=0.05,\;0.1$, and 0.2), some liposomes exhibited “Multiple blebs” (Fig. 5g, **Movie S13**), reminiscent of the morphological transition observed during apoptosis^5^. This is a distinct feature from the 2D network conditions (Fig. 2d, Fig. 3g).

Furthermore, as demonstrated in Fig. 3 and Fig. 4, we systematically investigated the individual contributions of $R_{C}$ and $R_{X}$ in the 3D network by varying $C_{H}$ and $C_{N}$ independently (Fig. S5). We confirmed that the basic two features revealed under the 2D network conditions (Fig. 3, Fig. 4) are preserved in the 3D network conditions: increasing $R_{C}$ shifted the dominant morphological transition mode from “No deformation” to “Weak deformation” and then to “Single bleb” (Fig. S5a,c), and increasing $R_{X}$ shifted the dominant bleb initiation mechanism from “Rupture” to “Detachment” (Fig. S5b,d). These parametric explorations further corroborate the universality of the regulatory mechanisms identified in the minimal reconstituted system. Moreover, we revealed that the 3D network with intermediate $R_{C}$ and $R_{X}$ is the optimal condition for multiple bleb formation (Fig. S5a,c).

### Catastrophic F-actin severing initiates bleb formation by the “Rupture” mechanism.

**Figure 6.**
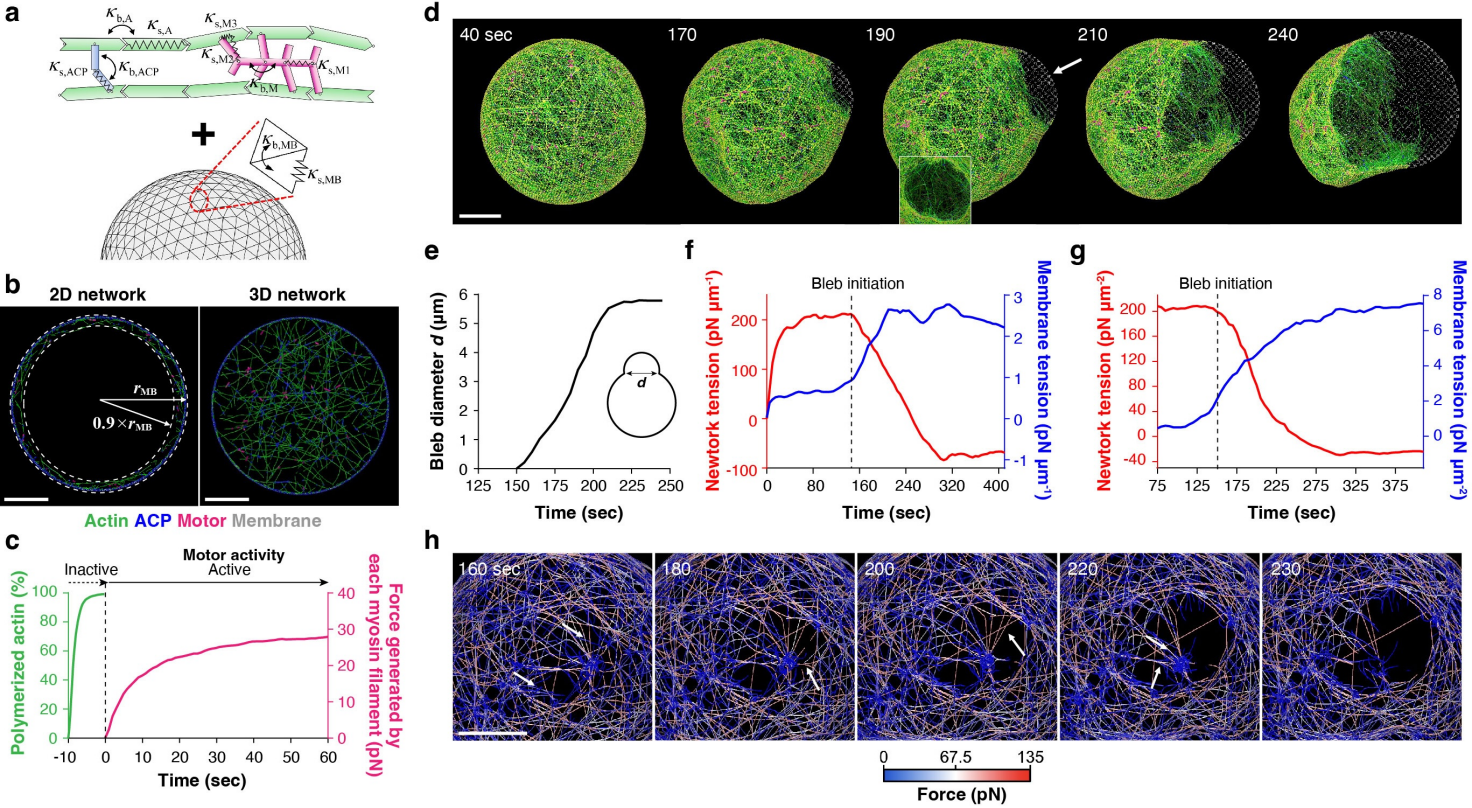
Agent-based computational model for dissecting the molecular mechanism of bleb formation and tension dynamics originating from the dynamic interplay of an actin network and a membrane. **a,** The model comprises an actin network encapsulated by a deformable membrane. **b,** Cross-sectional views of (left) 2D and (right) 3D networks assembled within the membrane. For the 2D network, the network was assembled near the membrane within a space between $r=0.9\times r_{MB}$ and $=r_{MB}$ , where $r_{MB}$ is a radial distance from the initial membrane center. The mean length of F-actin in the 2D network and 3D network was ~4.2 μm and ~5.2 μm, respectively. **c,** Time courses of actin polymerization and motor activity. These were estimated by measuring F-actin concentration and force generated by each myosin filament, respectively. Myosin motors were activated after network formation in an all-or-none fashion to reduce computational cost. This likely has minimal effects on network contraction and membrane deformation, as significant time was required to develop network tension before bleb initiation, as shown in **f. d,** Example of single bleb formation observed under the reference condition ($R_{X}=R_{C}=0.08$, motor density $R_{M}=0.01$) (**Movie S16**). The bleb started emerging from ~150 s in this example, followed by expansion. The inset shown at 190 s represents a hole on the network beneath the bleb in a different view as indicated by the arrow, showing that this bleb was initiated by a network rupture. **e,** The diameter of the bleb measured over time. **f,** Global tension acting on the network (red) and the membrane (blue) over time. **g,** Local network and membrane tension measured near the bleb over time. **h,** Time-lapse images showing that catastrophic severing events of F-actins indicated by arrows led to the initial formation and expansion of a rupture on the network (**Movie S17**). Scale bars, 2 μm.

Although our theoretical model could predict which mechanism is likely to trigger bleb initiation depending on the two physical parameters, how bleb initiation occurs at a molecular scale remains elusive. To define the molecular mechanism, we developed an agent-based model with an actin network encapsulated by a deformable membrane (Fig. 6a, Supplementary Note 2, Sections 1–8). The network is composed of F-actin, myosin mini-filaments, and actin crosslinking proteins (ACPs) with the geometry analogous to α-actinin. A fraction of ACPs can reversibly bind to the membrane as His-α-actinin used in experiments. The actin concentration was 10 μM in all simulations to be consistent with experiments. Unless specified, the membrane diameter was 8 μm, smaller than the mean diameter used in experiments (~20 μm) to reduce the computational cost. We confirmed that the results obtained using a system with a diameter of 16 μm were similar (Fig. S6, Fig. S7, **Movie S15**). As in the experiments, we created both a 2D cortical network (Fig. 6b, left) and a volume-spanning 3D network (Fig. 6b, right) and then compared the morphological transition process.

First, we performed simulations using the 2D network (Fig. 6b, left). Initially, actin nucleation and polymerization took place beneath the membrane in the presence of ACPs and myosin filaments to let them self-organize into the 2D network (Fig. 6c, green line). After the cortex formation, myosin was activated to start generating forces (Fig. 6c, magenta line). In the reference case with $R_{X}=R_{C}=0.08$, which is close to 1 μM His-α-actinin condition ($R_{X}=R_{C}=0.1$) in the experiments (Fig. 2), a single bleb started forming at ~150 s and then expanded substantially over time (Fig. 6d,e, Fig. S6a–d, **Movie S16**) as observed in the experiments. This bleb was initiated by a network rupture triggered by successive F-actin severing events induced by large tensile forces (Fig. 6h, Fig. S8a, **Movie S17**). Such rupture-initiated bleb formation was not observed when we did not incorporate F-actin severing in the model, indicating that severing is the essential process for the network rupture^53^ (Supplementary Note 2, Section 5).

The computational model enables us to measure the network tension and membrane tension separately. Global network tension increased and remained at a plateau (~200 pN μm^−1^) for ~70 s, after which it began to decrease upon bleb formation and expansion (Fig. 6f, red line, Fig. S6e,f). This plateau phase implies that it took significant time for large tensile forces to accumulate in a few filaments and reach the critical severing threshold^54^ (assumed to be 300 pN; Supplementary Note 2, Section 4). In contrast, global membrane tension was initially low and then increased during bleb expansion (Fig. 6f, blue line), as the expanding bleb membrane experienced expansile forces resulting from volume conservation. Local network/membrane tension measured near the bleb showed similar tendencies before and after bleb formation (Fig. 6g). The local network tension and local motor density were higher in the region showing bleb formation than those in the region showing only membrane deformation (Fig. S9a,b), implying that locally higher contractility induced by more motors enhanced network tension and thus led to the network rupture. This was verified by examining the impacts of motor density; higher motor density induced more frequent F-actin severing events and more blebs (Fig. S10a–c). The network rupture was also accompanied by a decrease in local membrane-bound ACP density (Fig. S6g, Fig. S9c–e). Taken together, the computational simulation revealed that catastrophic F-actin severing events at a local region are required for initiating bleb formation via the network rupture mechanism, and this is triggered by higher local motor density, followed by a local decrease in membrane-bound ACPs.

### Bleb is initiated by the “Detachment” mechanism when actin-membrane coupling is weak.

**Figure 7.**
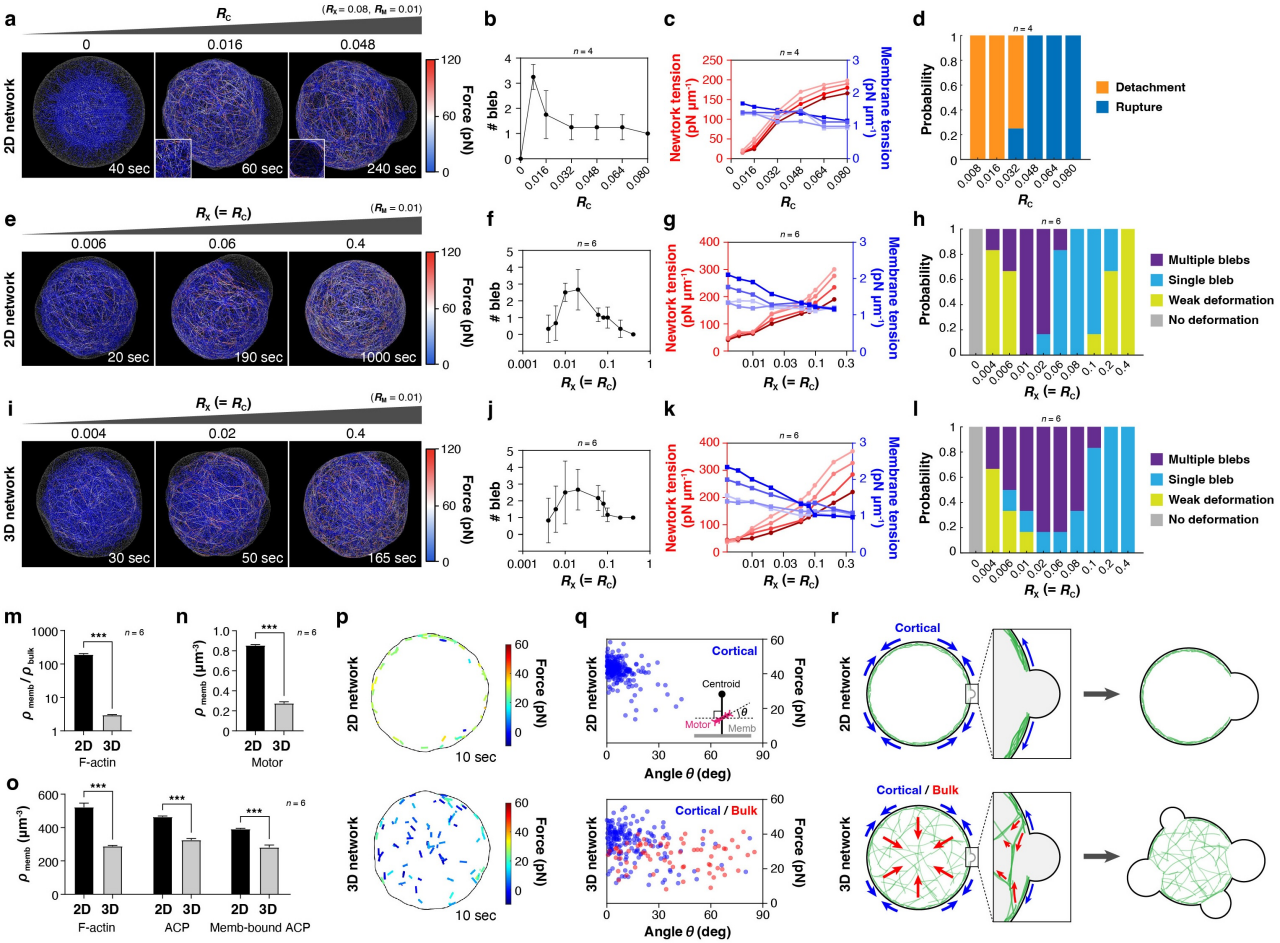
Different patterns of shape changes and bleb formation emerged, depending on the actin-membrane coupling strength, network connectivity, and the network dimensionality. **a-d,** Effects of actin-membrane coupling strength, *$R_{C}$* with the 2D network. $R_{C}$ was changed with $R_{X}$ fixed at the reference value (0.08). **e-h,** Effects of membrane-bindable ACP density (i.e., $R_{X}=R_{C}$) with the 2D network. **i-l,** Effects of membrane-bindable ACP density with the 3D network. **a,e,i,** Representative snapshots of the network and membrane under different conditions (**Movies S19-S24**). Color indicates force magnitude on each F-actin. (**a**, left) Without actin-membrane coupling ($R_{C}=0$), the network contracted into a small cluster without noticeable membrane deformation. (**a**, center) At $R_{C}=0.016$, bleb formation was initiated by detachment of the network from the membrane, evidenced by the intact network remaining beneath the bleb (inset). (**a**, right) At $R_{C}=0.048$, bleb formation was initiated by network rupture, evidenced by a hole in the network beneath the bleb (inset). **b,f,j,** Number of blebs as a function of (**b**) $R_{C}$ and (**f,j**) $R_{X}\left(=R_{C}\right)$. Error bars indicate SDs. **c,g,k,** Global network/membrane tension before and after bleb formation as a function of (**c**) $R_{C}$ and (**g,k**) $R_{X}\left(=R_{C}\right)$. Darker colors show tension at later times. **d,** Effect of $R_{C}$ on the bleb initiation mechanism. **h,l,** Effect of membrane-bindable ACP density ($R_{X}=R_{C}$) on morphological transition modes, with the (**h**) 2D or (**l**) 3D networks. **m,** Ratio of F-actin density near the membrane ($\rho_{\text{memb}}$) to F-actin density in the bulk ($\rho_{\text{bulk}}$) in the 2D and 3D networks under the reference condition ($R_{X}=R_{C}=\;0.08$, $R_{M}=0.01$). **n,** Density of motors beneath the membrane in the 2D and 3D networks ($R_{X}=R_{C}=\;0.08$, $R_{M}=0.01$). **o,** Densities of F-actin, ACP, and membrane-bound ACPs beneath the membrane in the 2D and 3D networks ($R_{X}=R_{C}=\;0.08$, $R_{M}=0.01$). **p,q,** Orientation of myosin filaments and the magnitude of motor forces in the 2D network (**Movie S25**) and the 3D network ($R_{X}=R_{C}=\;0.08$, $R_{M}=0.01$) (**Movie S26**) (**p**) visualized at a single time point and (**q**) the quantitative analysis results. **r,** Schematic representation of the force distribution in the 2D and 3D networks. Arrows indicate force directions, and the lengths represent magnitudes.

Using this 2D network model, we next examined the effect of the actin-membrane coupling $R_{C}$ by reducing it from the reference case, with the network connectivity $R_{X}$ fixed at 0.08 (Fig. 7a–d, Fig. S10d). Without network-membrane coupling ($R_{C}=0$), the network contracted into a smaller cluster without noticeable membrane deformation (Fig. 7a, left, Fig. S10d, left, **Movie S18**). At low network-membrane coupling ($R_{C}=0.008$ or 0.016), a bleb was initiated by the detachment of the network from the membrane (Fig. 7a, center, Fig. S10d, center, **Movie S19**). With $R_{C}$ higher than 0.048, bleb formation was driven by the network rupture as in the reference case (Fig. 7a, right, Fig. S10d, right, **Movie S20**). At the intermediate regime ($R_{C}=0.032$), some blebs were induced by “Detachment”, but the rest was induced by “Rupture” (Fig. 7d). These results are consistent with the experiments (Fig. 4b, left). We found that the network tension increased nearly proportional to $R_{C}$ up to the intermediate regime ($R_{C}=0.048$) (Fig. 7c, red lines), whereas membrane tension remained constant regardless of $R_{C}$ (Fig. 7c, blue lines). At higher $R_{C}$, robust coupling between the membrane and network enhanced the network stability. Thereby, a greater force was required to rupture the network and initiate bleb formation, consistent with the experiments (Fig. 3i, left, Fig. 3j, left). At lower $R_{C}$, the network began detaching from the membrane even before tension increased to a sufficiently high level required for F-actin severing (Fig. 7c, red lines, $R_{C}\leq 0.016$), resulting in bleb formation by the detachment mechanism, consistent with the model prediction (Fig. 4d, left).

### Comparison between 2D and 3D networks reveals an active role of the bulk actin network.

Our experiments showed that the 3D network induces the formation of multiple blebs more frequently (Fig. 2d, Fig. 5d, Fig. S5), implying the potential contribution of the sparse bulk actin network to the formation of multiple blebs. To dissect the underlying molecular mechanism, we ran simulations using both 2D and 3D networks (Fig. 6b) with various ACP densities and compared the outcomes (Fig. 7e–l). As in the case of experiments (Fig. 2, Fig. 5), all ACPs were allowed to bind to the membrane ($R_{X}=R_{C}$), so changes in $R_{X}$ also changed the density of actin-membrane coupling points.

With the 2D network in the absence of ACPs ($R_{X}=R_{C}=0$), neither the network contraction nor membrane deformation was observed (Fig. 7h). At low ACP density ($R_{X}=R_{C}=0.006$), either multiple small blebs or only membrane deformation without any bleb were observed (Fig. 7e, left, Fig. 7f,h, Fig. S10e, left, **Movie S21**). At intermediate ACP density ($R_{X}=R_{C}=0.06$), only a single bleb appeared more likely than multiple blebs (Fig. 7e, center, Fig. 7f,h, Fig. S10e, center, **Movie S22**). At high ACP density ($R_{X}=R_{C}=0.4$), blebs were negligibly small, which is attributed to high network connectivity (Fig. 7e, right, Fig. 7f,h, Fig. S10e, right, **Movie S23**); although small network ruptures followed by the emergence of tiny membrane bulges smaller than 0.8 μm in diameter were observed, they did not expand enough to initiate bleb formation because the ruptures did not increase in size due to a large number of cross-linking points between F-actins (Fig. S8b). While the initial membrane tension was rather independent of ACP density (Fig. 7g, blue lines), network tension increased nearly proportionally with ACP density, as more ACPs rendered the network more elastic by increasing both cross-linking points within the network and coupling points to the membrane (Fig. 7g, red lines).

Results obtained with the 3D network (Fig. 6b, right, Fig. 7i–l, Fig. S10f) were qualitatively similar to those obtained using the 2D network (Fig. 7e–h, Fig. S10e). The minimal ACP density required for the bleb formation was comparable between 2D and 3D networks ($R_{X}=R_{C}=0.004$), whereas the maximum ACP density permitting bleb formation was higher in the 3D network (Fig. 7h,l). In the 3D network, membrane-bindable ACPs were distributed across the entire volume within the membrane, resulting in fewer effective coupling points between the network and the membrane. Consequently, the bleb formation probability was higher in the 3D network at the same $R_{X}\left(=R_{C}\right)$. We compared the densities of F-actin, myosin motors, and membrane-bound ACPs between the 2D and 3D networks for our reference case ($R_{X}=R_{C}=0.08$), and confirmed that all of these components were present at lower densities beneath the membrane in the 3D network (Fig. 7m–o), consistent with the F-actin distribution observed in the experiments (Fig. 2c, Fig. 5c, Fig. S2e).

Importantly, with the 3D network, multiple blebs formed over a wider range of ACP densities (Fig. 7h,l, purple bars and **Movie S24**), reproducing the trend observed in the experiments (Fig. 2d, Fig. 4d). We found that the orientation angle of individual myosin filaments showed a marked difference between 2D and 3D networks, and not only the cortical myosin filaments but also myosin filaments in the bulk region generated significant forces (Fig. 7p,q, **Movie S25, Movie S26**). While myosin filaments beneath the membrane were oriented parallel to the membrane, which may contribute to the cortical tension development both in 2D and 3D networks (Fig. 7q, blue plots), myosin filaments in the bulk region of the 3D network showed nearly random orientations (Fig. 7q, red plots), which can generate inward pulling force to the membrane as an ensemble (Fig. 7r, left). At the molecular scale, sparse bundled actin networks in the bulk region generate contractile forces that act as antagonistic forces opposing membrane expansion (Fig. 7r, insets). This will trigger the formation of new blebs at different positions to release the elevated pressure in liposomes, resulting in multiple bleb formation more likely (Fig. 7r, right). Overall, although the cortical network is expected to play a central role in bleb formation, our results indicate that the bulk actin network may also play an important role in regulating the number of blebs in cells.

### Local perturbation on actin-membrane coupling strength or network connectivity controls bleb position.

**Figure 8.**
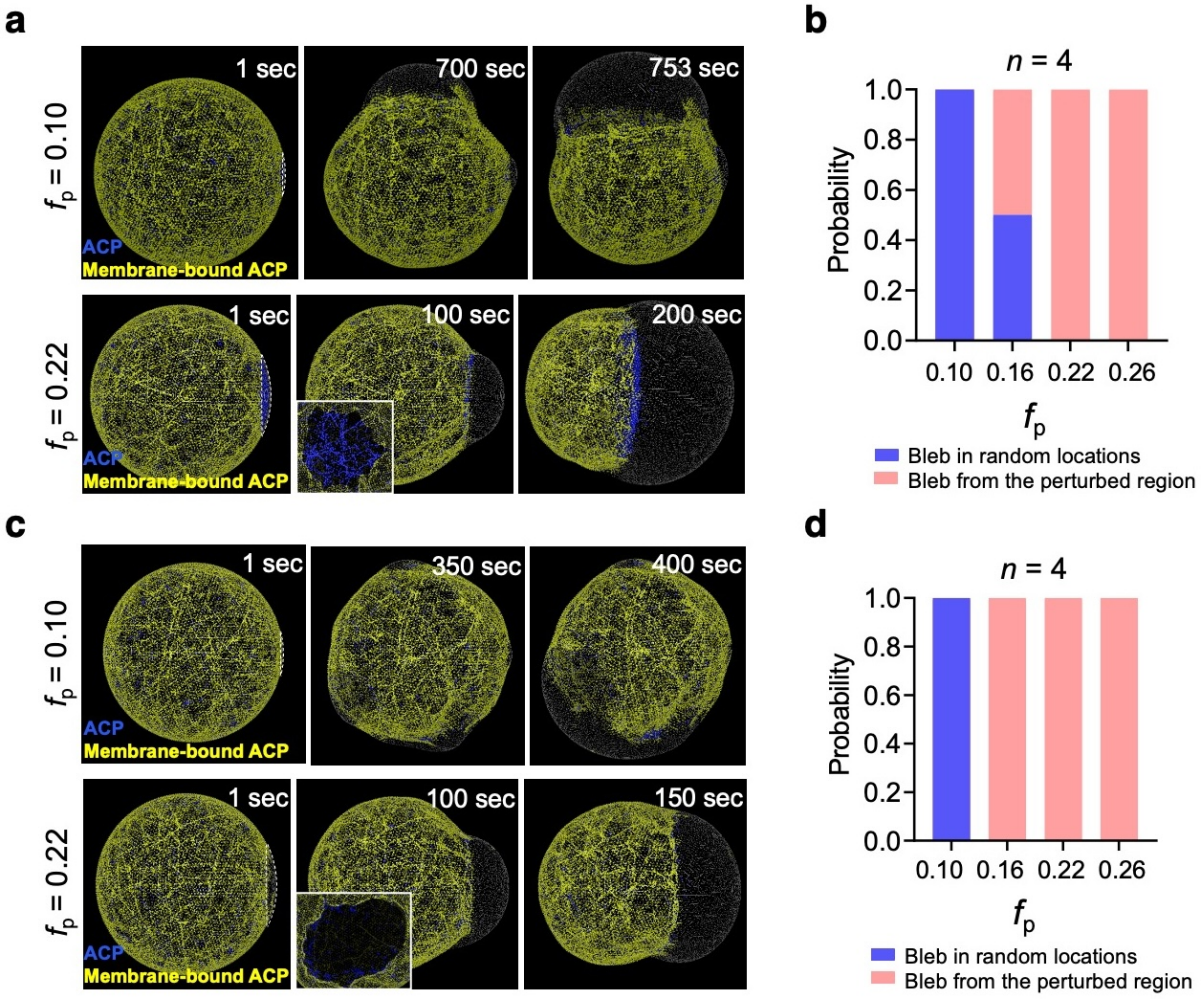
Local perturbation on actin-membrane coupling strength or network connectivity controls bleb position. **a,b,** Perturbation on actin-membrane coupling strength, $R_{C}$. **c,d,** Perturbation on network connectivity, $R_{X}$. We tested whether a local perturbation could initiate bleb formation from a specified location by selectively preventing ACPs from binding to the membrane ($R_{C}=0$) or binding to F-actin ($R_{X}=0$) within a region defined by a fraction of the membrane surface, $f_{p}$ (white dashed lines in **a** and **c**; t=1sec), where $f_{p}$ indicates ratio of the perturbed membrane area to the total membrane area. **a,** With $f_{p}=0.10$ (top), a bleb formed in a different location. With $f_{p}=0.22$ (bottom), a bleb emerged from the perturbed region, initiated by the detachment of the network from the membrane, as evidenced by the intact network remaining beneath the bleb (inset). **c,** With $f_{p}=0.10$ (top), a bleb formed in a different location. With $f_{p}=0.22$ (bottom), a bleb emerged from the perturbed region, initiated by the rupture of the network, as evidenced by a hole in the network beneath the bleb (inset). **b,d,** Dependence of $f_{p}$ on the probability of bleb formation from the perturbed region. At $f_{p}=0.16$, perturbation on $R_{C}$ controlled the bleb position with 50% efficiency (**b**), whereas perturbation on $R_{X}$ controlled the bleb position with 100% efficiency (**d**). This difference arises because ACPs contribute to both actin-membrane coupling and network connectivity. In the absence of ACPs ($R_{X}=0$), the network becomes mechanically weaker, making the system more susceptible to bleb formation.

Previous studies in living cells^55^ and reconstituted systems^20^ have demonstrated that local laser ablation of the actin cortex can trigger bleb formation. However, the key molecular parameters within the actin cortex that govern bleb initiation remain unclear, because laser ablation disrupts the actin network nonspecifically. In this study, we have identified both *in vitro* and *in silico* that the actin-membrane coupling strength, $R_{C}$, and the network connectivity, $R_{X}$, are the key parameters regulating bleb formation and the initiation mechanism. Using the *in silico* model, we tested whether external perturbation, either on $R_{C}$ or $R_{X}$, can induce bleb formation in a specified position. Specifically, we locally reduced $R_{C}$ (Fig. 8a,b) or $R_{X}$ (Fig. 8c,d) to different extents under the reference condition where bleb formation via “Rupture” was observed. When the perturbation level was low, a bleb was still formed at random positions. When the perturbation level was sufficiently high, the bleb was consistently formed from the perturbed region. Interestingly, we could switch the mechanism from “Rupture” to “Detachment” by inducing decoupling of a large portion of the cortex from the membrane (Fig. 8a). These simulations clarified that the location and mechanism of bleb formation can be controlled by locally manipulating one of the two parameters.

## DISCUSSION

The actin cortex is a ubiquitous structure found in various cells. However, the interaction between actomyosin machinery and the upstream/downstream signaling pathways, which are different between cell types and species, makes it difficult to isolate the contribution of actomyosin machinery to the morphological transition process. In this study, we combined an *in vitro* and *in silico* systems (Fig. 1, Fig. 6) to dissect how and to what extent actomyosin machinery contributes to the regulation of membrane deformation without any biochemical signaling cue.

We demonstrated that the single parameter, His-α-actinin concentration $C_{H}$, can switch the morphological transition mode of cell-sized liposomes (Fig. 2, Fig. 5, Fig. 7e–l). Interestingly, with high actin-membrane coupling strength and high network connectivity with the 3D network organization, the minimal model system in both experiments and simulations always formed a single bleb (Fig. 5d, Fig. 7l). This result indicates that the actin cytoskeleton can form cell-scale polarity solely by the mechanics of cytoskeletal proteins and the plasma membrane, even in the absence of spatial biochemical cues. At the initial stage of cell migration mediated by the actin cortex, the cell spontaneously establishes cell-scale polarity to define the front- and rear-end by changing uniform actin cortex to a polarized actomyosin structure^3,5^. Similar polarization is observed in the one-cell stage of *C. elegans* embryos, in which asymmetric actin network contraction segregates PAR polarity proteins to define the anterior-posterior axis^55^. Our findings suggest that the cell tunes the actin network connectivity and cortex-membrane attachment to ensure the robust formation of cell polarity.

There is an ongoing debate about the bleb initiation mechanism^2^. Observations in various cells suggest that the bleb initiation process may involve two distinct mechanisms: the local rupture of the actin cortex^13,57^ or detachment of the membrane from the actin cortex^58^. The mechanism seems to depend on the cell types and animal species, and thus a comprehensive understanding of the two distinct bleb initiation mechanisms is still missing. In particular, it remains unclear which specific parameters determine the switch between these two mechanisms. Here, we demonstrated both *in vitro* and *in silico* that the two mechanisms can be switched by varying the actin-membrane coupling strength $R_{C}$ and network connectivity $R_{X}$ (Fig. 4, Fig. 7a–d). The physical model (Fig. 4c–e) in combination with simulations (Fig. 6, Fig. 7a–d) clarified that the bleb initiation mechanism depends on whether adhesion between the actin cortex and the membrane is sufficient to sustain myosin-induced forces until F-actin in the network undergoes catastrophic rupture severing by strong tensile forces.

Control of the bleb initiation mechanism is important for regulating cell functions. Recent observations on cancer cells^59^ and primordial germ cells^60^ have revealed that the endoplasmic reticulum (ER) network in the cytoplasm flows into the expanding bleb. This ER network entry stimulates store-operated calcium entry (SOCE)-mediated calcium flux to the expanding bleb through the formation of the STIM/Orai complex between the ER and the plasma membrane. When the bleb is initiated by the detachment mechanism, the remaining cortex may serve as a physical barrier that blocks ER network entry into the expanding bleb. During bleb-based motility, the remaining cortex can prevent the nucleus and other organelles from being translocated to the leading edge. In this study, we showed that the rupture mechanism is preferred in the presence of strong actin-membrane coupling. Previously, we demonstrated using cell-extract-encapsulated droplets that strong actin-membrane coupling is the key factor for transmitting the force generated by the actomyosin machinery to extracellular environments for motility^61^. Therefore, from a physical viewpoint, it is reasonable for motile cells to select the rupture-initiated bleb formation for efficient force transmission. Thus, the two distinct initiation mechanisms likely have specific biological roles, and tight regulation of the bleb initiation process may be crucial for controlling cell functions.

Control of the bleb position is also important. For example, E-cadherin confines the bleb-forming region to a restricted area at the leading edge of migrating cells by exerting frictional forces to impede the backward flow of actomyosin structures^62^. This localized bleb formation is crucial for maintaining the front-rear polarity and directional persistence during migration. In this study, using simulations, we showed local reduction of actin-membrane coupling strength or network connectivity can induce bleb formation at the perturbation site (Fig. 8). These observations imply that cells control bleb positions by locally manipulating one of the two physical parameters by ph;osphorylations^63,64^.

Furthermore, we successfully reconstituted the formation of multiple blebs from a minimal set of cytoskeletal elements. A combination of experiments and simulations revealed the active role of the bulk actin network in the formation of multiple blebs (Fig. 2, Fig. 5, Fig. 7i–r, Fig. S5). However, the parameter range for the multiple bleb formation was narrow. Indeed, in *in vitro* experiments, multiple blebbing was not a predominant event even under optimal conditions, compared to the single bleb formation, which was robust and occurred with high probability (Fig. 2, Fig. 5, Fig. S5). This suggests that additional factor(s) may be required to enable the robust formation of multiple blebs. In cells, the actin network in the bulk cytoplasm is physically linked with the microtubule^65^ and intermediate filament^66^ networks. Future studies will be needed to examine how these composite network organizations contribute to the regulation of bleb number. In addition, the cytoplasm in living cells is considerably more viscoelastic and heterogeneous than in our reconstituted system^67,68^. We confirmed that the encapsulated buffers were one order of magnitude smaller than the viscosity of cytoplasm^69,70^ (Materials and Methods). Therefore, pressure release after bleb initiation is likely to propagate rapidly throughout the liposome^24^, thereby suppressing the formation of additional blebs in our reconstituted model. A key future challenge will be to investigate how the viscoelastic properties and spatial heterogeneity of bulk cytoskeletal networks affect the number and position of blebs both *in vitro* and *in vivo*.

In summary, a combination of *in vitro* reconstitution experiments and agent-based modeling has elucidated the extent to which the mechanical properties of the actomyosin network can control the morphological transitions of cell-sized liposomes, providing physical insights into how the cell tunes actin-membrane coupling strength, network connectivity, and actin distribution to control cell shape and biological functions.

## MATERIALS AND METHODS

### Buffer.

All the experiments were performed using A50 buffer (50 mM HEPES-KOH pH 7.6, 50 mM KCl, 5 mM MgCl_2_, 1 mM EGTA)^71,72,73^, unless stated separately.

### Protein preparation.

Actin was purified from rabbit white skeletal muscle as previously described^74^, snap-frozen in liquid nitrogen, and stored at −80°C in G buffer (2 mM Tris-HCl, pH 8.0, 0.05 mM CaCl_2_, 2 mM NaN_3_, 0.1 mM ATP, 0.5 mM 2-mercaptoethanol). Unphosphorylated smooth muscle myosin (SMM) was purified from chicken gizzards following our previous protocol^73^ and labeled with Alexa Fluor 488 NHS ester (A20000, Thermo) as needed. The samples were snap-frozen in liquid nitrogen and stored at −80°C in A50 buffer containing 1 mM DTT and 1 mM ATP. Recombinant human ZIP kinase (ZIPK, GST-tagged) was prepared as previously described^73^, snap-frozen in liquid nitrogen, and stored at −80°C in A50 buffer containing 1 mM DTT. Recombinant human α-actinin I (6×His-tagged) was prepared according to our previous report^72^. The His-tag was removed using PreScission protease (27084301, Cytiva) and labeled with Alexa Fluor 488-maleimide (A110254, Thermo) as needed. The samples were snap-frozen in liquid nitrogen and stored at −80°C in A150 buffer (50 mM HEPES-KOH, pH 7.6, 150 mM KCl, 5 mM MgCl_2_, 1 mM EGTA) supplemented with 1 mM 2-mercaptoethanol.

The purity of proteins was confirmed by SDS-PAGE (Fig. S1a). The concentrations were determined from UV absorbance, using absorption coefficients: $A_{290\;nm}^{1\%}=6.3{\;cm}^{-1}$ for G-actin, $A_{280\;nm}^{1\%}=10.84{\;cm}^{-1}$ for his-tagged α-actinin I, and 9.61 cm^−1^ for his-tag removed α-actinin I, and molecular weights of 42,000 Da for G-actin, 105,300 Da for his-tagged α-actinin I, and 100,000 Da for his-tag removed α-actinin I. The other protein concentrations were determined using Protein Assay Kit (500–0006, Bio-Rad), and molecular weights of 474,000 Da for SMM (hexamer: 2 myosin heavy chains (MHC) + 2 myosin regulatory light chains (MRLC) + 2 myosin essential light chains (MELC)), and 79,084 Da for ZIPK.

### Preparation of lipid-oil mixture.

The lipid-oil mixture was prepared as previously described^71^. Briefly, L-α-phosphatidylcholine from chicken egg yolk (egg PC; 840051P, Avanti Polar Lipids), 1,2-dioleoyl-sn-glycero-3-phosphatidylglycerol (DOPG; 840475P, Avanti Polar Lipids), and 1,2-dioleoyl-sn-glycero-3-[(N-(5-amino-1-carboxypentyl)iminodiacetic acid)succinyl] (nickel salt) (DGS-NTA(Ni); 790404C, Avanti Polar Lipids) were mixed at a molar ratio of 85:10:5 and then dissolved in mineral oil (M8410, Sigma-Aldrich) using a bath sonicator at 60°C and the power of 60 W for 90 min. The total lipid concentration in oil was 5 mM.

### Protein encapsulation into liposomes.

The inverted emulsion method^75^ was used to encapsulate purified cytoskeletal proteins into cell-sized liposomes^71^. Prior to the experiments, 100–150 μl of the lipid-oil mixture was gently placed on 1 ml of the outer solution (A50 buffer containing 110 mM glucose, 2 mM Trolox, 10 mM DTT, and 0.2 mg ml^−1^ BSA) in a 1.5 ml sample tube, and incubated on ice for >5 min to allow for the assembly of lipid monolayer at the oil/outer-solution interface. Next, 1.5 μl of ×20 E-mix (20 mM ATP, 200 mM phosphocreatine, 2 mg ml^−1^ creatine phosphokinase, 200 mM DTT) in A50 buffer was mixed with 1.5 μl of 10 μM Alexa Fluor 546-conjugated phalloidin (A22283, Thermo) in ×3 A0 + KLC50 buffer (150 mM HEPES-KOH pH 7.6, 50 mM KCl, 15 mM MgCl_2_, 3 mM EGTA), 3 μl of 1 M sucrose and 20 mM Trolox in A50 buffer, 6 μl of 1% (w/v) methylcellulose (1,500 cP; 135–02142, Wako) in A50 buffer, 3 μl of α-actinin I in A150 buffer, and 3 μl of SMM in A50 buffer. Then, 3 μl of ZIPK in A50 buffer was added to initiate myosin phosphorylation. Within a few seconds, this mixture (27 μl) was added to 3 μl of 100 μM G-actin in G buffer and pipetted 5 times to mix instantly. The actin polymerization was initiated by this mixing, and this timing was defined as t=0 sec. Subsequently, 2 μl of the mixture was put into 100 μl of the lipid-oil mixture pre-incubated on ice in a 1.5 ml sample tube, and this sample was vortexed for 10–15 sec to form cell-sized droplets. The emulsion was gently placed on the lipid-oil mixture placed on the outer-solution, and then the tube was centrifuged at 12,000 ×*g* for 60 sec at 4°C to transform droplets into liposomes. In each experiment, the rest of the protein mixture (28 μl) was subjected to the measurement of the osmotic pressure by using a micro-osmometer (Fiske 210, Advanced Instruments). It was confirmed that the pressure difference between the encapsulated protein solution and the outer medium was smaller than ±17 mOsm (less than 5% difference between the inner and outer solutions; the mean difference was −0.23 mOsm, n=222) to reasonably approximate that the liposomes were settled at isotonic condition.

An observation chamber was assembled by placing two double-sided tapes (0.3 mm in thickness) onto a coverslip (60 × 24 mm^2^, thickness No.1, Matsunami) with a coverslip (18 × 18 mm^2^, Matsunami) on top. Immediately after the liposome formation, the liposome solution was perfused into the chamber and sealed with Valap to prevent flow and then warmed to 25 ± 1°C to promote actin polymerization and myosin phosphorylation. The inner surface of the chamber was immediately coated with BSA, which was included in the outer solution, to prohibit non-specific interaction between the liposomes and the coverslips. Note that we added 100 mM of sucrose inside the liposomes and used the equimolar concentration of glucose for the outer solution to create a mass density difference to settle down the liposomes on a bottom coverslip for time-lapse imaging. We confirmed that this mass density difference was enough to sediment the liposomes within 10 min after making an observation chamber and was small enough to keep the liposomes spherical.

The liposomes contained 1 mM ATP, 10 mM phosphocreatine, 0.1 mg ml^−1^ of creatine phosphokinase, 100 mM sucrose, 2 mM Trolox, 10 mM DTT, 0.2% or 0% (w/v) methylcellulose (1,500 cP), 10 μM actin, 0.5 μM Alexa Fluor 546-conjugated phalloidin, 1 μM SMM, and various concentrations of α-actinin I (± his-tag), and 1.4 × 10^−4^ unit ml^−1^ ZIPK. One unit is defined as the amount of ZIPK that phosphorylates 1 μ mol of MRLC in SMM per minute at 25°C. We have reported that 5% (mol/mol) of Alexa Fluor 546-conjugated phalloidin does not change the actin polymerization kinetics, monitored by pyrene-assay^72^.

### Microscopy.

Epifluorescence and bright-field images of the liposomes were observed by a custom-built inverted microscope equipped with a ×60 objective (PlanApo NA1.45 oil TIRFM or PlanApo NA1.40 oil, Olympus), an electron-multiplying CCD (charge-coupled device) camera (iXon3 DU-897E-CS0-#BV, Andor Technology). Confocal fluorescence images of the liposomes were observed by an inverted microscope (IX73, Olympus) equipped with a ×60 objective (UPlanSApo NA 1.30 sil, Olympus), a confocal scanner unit (CSU10, Yokogawa), an electron-multiplying CCD camera (iXon3 DU-897E-CS0-#BV, Andor Technology). For both microscopies, a custom-built heat block connected to a water bath (AB-1600, ATTO) was mounted on the objective lens to maintain the sample temperature at 25 ± 1°C.

### Analysis of the distribution of F-actin in liposomes.

To quantify the steady-state distribution of F-actin, myosin and ZIPK were excluded from the inner solution of liposomes to eliminate the membrane deformation and contraction of actomyosin networks. The liposomes were observed after >20 min from protein encapsulation, in which time scale it was reasonable to assume a steady state with a balance between polymerization and depolymerization reactions, considering net actin polymerization was almost terminated (Fig. 1c). Using confocal microscopy, we measured F-actin density beneath the membrane and compared the value with F-actin density in the bulk region of the liposome (Fig. 2b,c, Fig. 3b,c, Fig. 5b,c, Fig. S2b–e).

### Observation of myosin filaments and the length analysis.

One micromolar of unphosphorylated SMM labeled with Alexa Fluor 488 (10% labeling ratio) was incubated with 1.4 × 10^−4^ unit ml^−1^ ZIPK in A50 buffer containing 100 mM sucrose, 2 mM Trolox, 0.05 mg ml^−1^ BSA, 1 mM ATP, 10 mM phosphocreatine, 0.1 mg ml^−1^ creatine phosphokinase, and 10 mM DTT at 25°C (ThermoMixer C, Eppendorf) for 120 min in a 1.5 ml sample tube to induce mini-filament formation. BSA was included in the reaction mixture to prevent non-specific adsorption of ZIPK to the tube surface. After the incubation, the sample was immediately diluted 100-fold with the same buffer without ZIPK and BSA. A 4 μl aliquot of the diluted sample was placed onto a coverslip (60 × 24 mm^2^, thickness No.1, Matsunami), covered with a smaller coverslip (18 × 18 mm^2^, Matsunami), and sealed with Valap. The sample was observed using a custom-built total internal reflection fluorescence (TIRF) microscope equipped with a ×60 objective (UPlanApo NA 1.50 oil, Olympus) and EM-CCD camera (iXon3 DU-897E-CS0-#BV, Andor Technology) (Fig. 1e). Filament length was measured using fluorescence images (Fig. S1b). After background subtraction using a rolling-ball algorithm, binary images were generated, and the length of the major axis of each particle was measured. Overlapping filaments were manually excluded from the analysis.

### Analysis of the phosphorylation rate of myosin.

The phosphorylation rate of SMM by ZIPK was measured using urea/glycerol PAGE^35^. One micromolar of unphosphorylated SMM was incubated with 1.4 × 10^−4^ unit ml^−1^ or 0.53 × 10^−4^ unit ml^−1^ ZIPK in A50 buffer containing 100 mM sucrose, 2 mM Trolox, 0.05 mg ml^−1^ BSA, 1 mM ATP, 10 mM phosphocreatine, 0.1 mg ml^−1^ creatine phosphokinase, and 10 mM DTT at 25°C (ThermoMixer C, Eppendorf) for 120 min (1.4 × 10^−4^ unit ml^−1^ ZIPK) or 240 min (0.53 × 10^−4^ unit ml^−1^ ZIPK) in a 1.5 ml sample tube to induce phosphorylation. BSA was included in the reaction mixture to prevent non-specific adsorption of ZIPK to the tube surface. The initial sample volume was 400 μl. Every 10, 20, or 40 min incubation, 30 μl of the reaction mixture was taken from the sample tube and the reaction was terminated by the addition of 1.1 g ml^−1^ urea. Unphosphorylated (0P), monophosphorylated (1P), and diphosphorylated (2P) myosin regulatory light chains (MRLCs) were separated by urea/glycerol PAGE (10 mA, 230 min). The gel was stained using the SYPRO Ruby protein gel stain (S12000, Invitrogen), and the band intensities were analyzed using Image Lab (Bio-Rad) (Fig. 1c,d, Fig. S4b,c).

### Analysis of the polymerization rate of actin.

The polymerization rate of actin was measured using a pyrene assay^34,72^. Briefly, 8 μl of 100 μM G-actin (5% pyrene-labeled) in G buffer was put into a 384-well microplate (242764, Thremo), then 72 μl of A50 buffer containing ATP, phosphocreatine, creatine phosphokinase, sucrose, Trolox, and DTT was added and pipetted 5 times. Then, the fluorescence intensity was measured using a microplate reader (Fluoroskan FL, Thermo) (Fig. 1c). The final concentrations were 10 μM actin, 1 mM ATP, 10 mM phosphocreatine, 0.1 mg ml^−1^ of creatine phosphokinase, 100 mM sucrose, 2 mM Trolox, and 10 mM DTT.

### Analysis of the binding affinity of His-α-actinin to F-actin.

To measure the binding affinity of His-α-actinin to F-actin, a co-sedimentation assay was performed^76^. Various concentrations of His-α-actinin in A50 buffer were mixed with 10 μM of G-actin on ice, incubated at 25°C for 60 min in a water bath (AB-1600, ATTO) to polymerize actin, then centrifuged at 100,000 ×*g* for 10 min at 25°C. Identical aliquots of the supernatant (S) and the pellet suspended in A50 buffer with the initial volume before centrifugation (P) were applied to SDS-PAGE. (5–20% gradient precast gel, ATTO), and the gel was stained by Coomassie Brilliant Blue (Fig. S1c). The fractions of bound His-α-actinin and free His-α-actinin were calculated from the band intensities^76^. The dissociation constant was determined from the model fitting^77^ (Fig. S1d).

### Measurement of the buffer viscosity.

The buffer viscosities were measured with Ostwald’s viscometer (φ=0.75mm; CL2370–02-10, Climbing). The measured viscosities at 24°C were 0.92 mPa s for A50 buffer and 2.27 mPa s for A50 buffer containing 0.2% (w/v) methylcellulose, both of which were one order of magnitude smaller than the viscosity of cytoplasm^69,70^.

### Agent-based model.

We developed an agent-based model for a cell-like structure consisting of an actin network and a membrane. The actin network consists of F-actin, actin cross-linking proteins (ACPs), and motors, all of which are simplified via cylindrical segments. The membrane is simplified into a triangulated mesh. The displacements of all points defining the segments and the triangulated mesh are updated at each time step via the Langevin equation with consideration of extensional, bending, and repulsive forces. F-actin is assembled via nucleation and polymerization events but do not undergo depolymerization. However, F-actin can be severed into two filaments if they experience a tensile force beyond a threshold level. ACPs connect pairs of F-actins to form cross-linking points, and motors bind to F-actin and then walk toward its barbed end. At the beginning of each simulation, an actin network is created via self-assembly and interactions of cytoskeletal elements with specified cross-linking density ($R_{X}$) and motor density ($R_{M}$) within a spherical membrane. In the 2D network simulations, a thin actin network is assembled only beneath a spherical membrane to create a cortical network (Fig. 6b, left). In the 3D network simulations, a network is assembled in an entire space within the membrane to create a 3D actin structure (Fig. 6b, right). During the network assembly, the spherical membrane does not undergo deformation, and the network and membrane are coupled via a fraction of ACPs defined by the extent of coupling. The fraction is varied between 0 and 1, and it is equal to $R_{C}/R_{X}$, where $R_{C}$ is actin-membrane coupling strength. Only after the network assembly, motors start walking to generate mechanical forces, and the membrane also starts deforming by thermal fluctuation and motor-generated forces. The details of the agent-based model and parameters used in simulations are explained in Supplementary Note 2.

### Analysis of the orientational angles of motors in simulations.

The angle between motor backbones and the circumferential direction was calculated as follows. First, an acute angle between a vector defined by the two ends of the motor backbones, ${\vec{r}}_{MB}$, and a vector defined from the membrane center to the centroid of the motor backbones, ${\vec{r}}_{OM}$, was calculated. Then, the angle was subtracted from 90°:

$$\theta_{MB}=90^{\circ }-{cos}^{-1}\left(\frac{\left|{\vec{r}}_{MB}\;\cdot {\vec{r}}_{OM}\right|}{\left|{\vec{r}}_{MB}\right|\left|{\vec{r}}_{OM}\right|}\right).$$

### Data analysis.

Image analysis was performed using Fiji (NIH) and MATLAB (MathWorks). Statistical analysis was performed using MATLAB (MathWorks).

## Supplementary Material

Supplement 1

## Data Availability

Data are available from the corresponding authors upon reasonable request.
