# Supplementary material for: Reconstitution of actomyosin networks in cell-sized liposomes reveals distinct mechanical roles of cytoskeletal organization in membrane shape remodeling
Source: bioRxiv. 2025 May 22:2025.05.18.654456. Preprint. [Version 1] doi: 10.1101/2025.05.18.654456 (PMC12139798; doi:10.1101/2025.05.18.654456)
Supplement: Supplement 1 [file NIHPP2025.05.18.654456v1-supplement-1.pdf]

bioRxiv preprint doi: https://doi.org/10.1101/2025.05.18.654456; this version posted May 22, 2025. The copyright holder for this preprint (which was not certified by peer review) is the author/funder, who has granted bioRxiv a license to display the preprint in perpetuity. It is made available under aCC-BY 4.0 International license.

SUPPLEMENTARY FIGURES

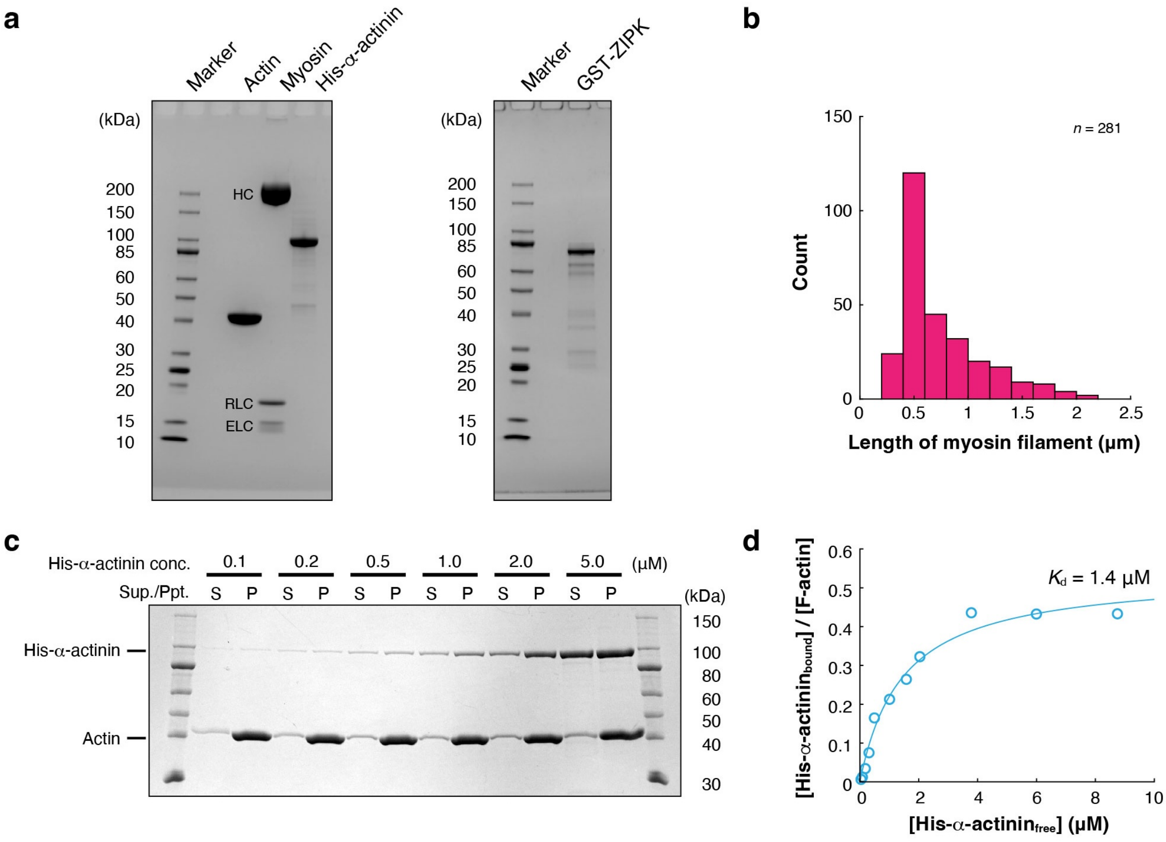

Figure S1. Biochemical properties of proteins used in reconstitution experiments.

a, Protein purity confirmed by SDS-PAGE. Cytoskeletal proteins (left) and ZIPK (right) used in the experiments were applied to 5-20% gradient polyacrylamide gel. The gels were stained by Coomassie Brilliant Blue. Smooth muscle myosin is a protein complex composed of heavy chain (HC), regulatory light chain (RLC), and essential light chain (ELC). **b,** Length distribution of myosin filaments. The fluorescence images of individual myosin filaments were analyzed. Submicrometer-long mini-filaments (0.64 ± 0.30 μm (mean ± SD, n = 550)) were formed by phosphorylation. **c,d,** Dissociation constant K_d of α-actinin estimated from co-sedimentation assay. **c,** Representative image of SDS-PAGE. Various concentrations of His-α-actinin were mixed with G-actin on ice, incubated at 25°C to polymerize actin, and then centrifuged to precipitate His-α-actinin-F-actin complex. Identical aliquots of the supernatant (S) and the pellet suspended in A50 buffer with the initial volume before centrifugation (P) were applied to 5-20% gradient polyacrylamide gel. The fractions of bound His-α-actinin and free His-α-actinin were calculated from the band intensities. **d,** Experimental data (circles) and the model (solid line): $y = B_{max}\, x / (K_d + x)$. The dissociation constant K_d = 1.4 μM was determined from the model fitting using K_d and B_{max} as the fitting parameters (B_{max} = 0.53).

bioRxiv preprint doi: https://doi.org/10.1101/2025.05.18.654456; this version posted May 22, 2025. The copyright holder for this preprint (which was not certified by peer review) is the author/funder, who has granted bioRxiv a license to display the preprint in perpetuity. It is made available under aCC-BY 4.0 International license.

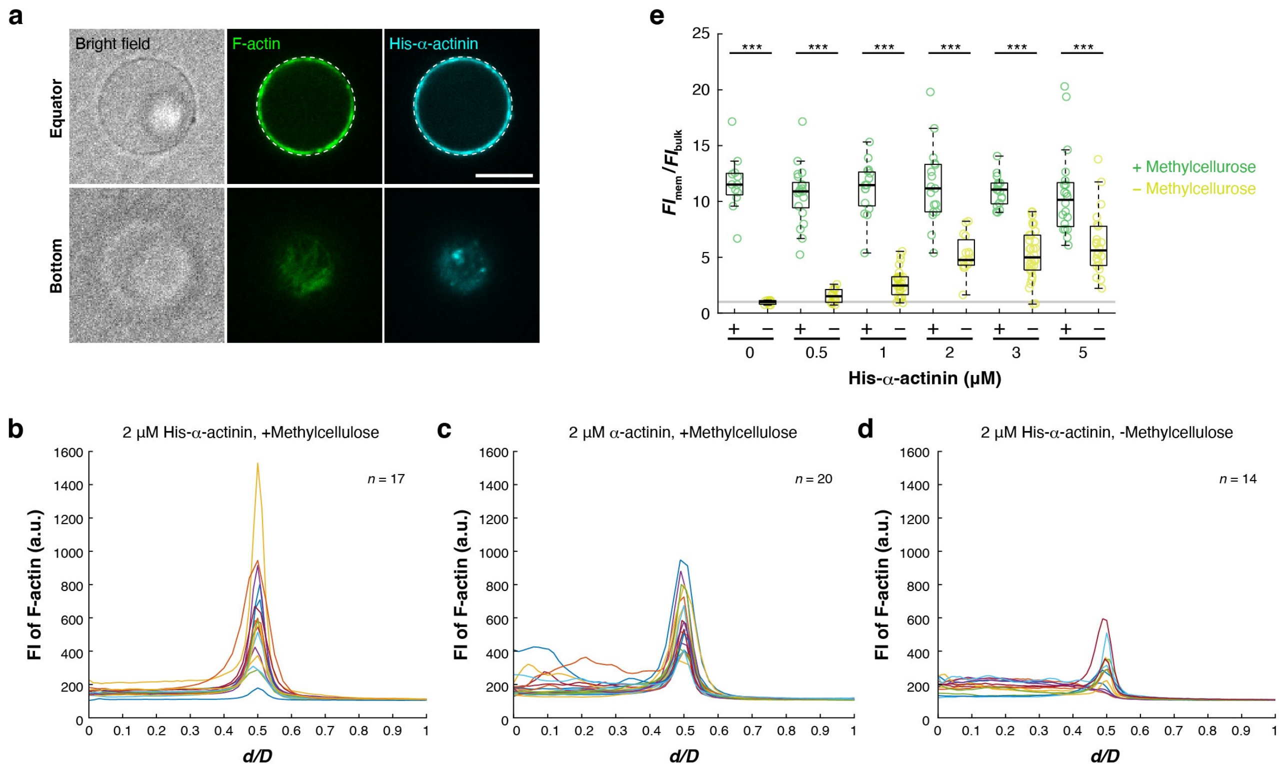

Figure S2. Distribution of proteins in liposomes.

a, A cross-sectional confocal image of a liposome containing 10 μM actin, 2 μM His-α-actinin (Alexa488-labeled), and methylcellulose at the equator. F-actin and His-α-actinin co-localized beneath the membrane, forming a 2D cortical network. F-actin was visualized using 5% (mol/mol) of Alexa546-phalloidin. The liposome did not contain myosin and ZIPK. White dashed lines indicate the liposome periphery. Scale bar, 10 μm. **b-d,** Fluorescence intensity profiles of F-actin along the radial direction of individual liposomes. Different colors represent different liposomes. **b**, 2 μM His-α-actinin in the presence of methylcellulose. **c,** 2 μM α-actinin in the presence of methylcellulose. **d,** His-α-actinin in the absence of methylcellulose. These are representative raw data from **Fig. 2c, Fig. 3c,** and **Fig. 5c**. **e,** Comparison of cortical F-actin densities between the presence and absence of methylcellulose. ***: $p < 0.001$ (Welch's t-test, two-sided).

bioRxiv preprint doi: https://doi.org/10.1101/2025.05.18.654456; this version posted May 22, 2025. The copyright holder for this preprint (which was not certified by peer review) is the author/funder, who has granted bioRxiv a license to display the preprint in perpetuity. It is made available under aCC-BY 4.0 International license.

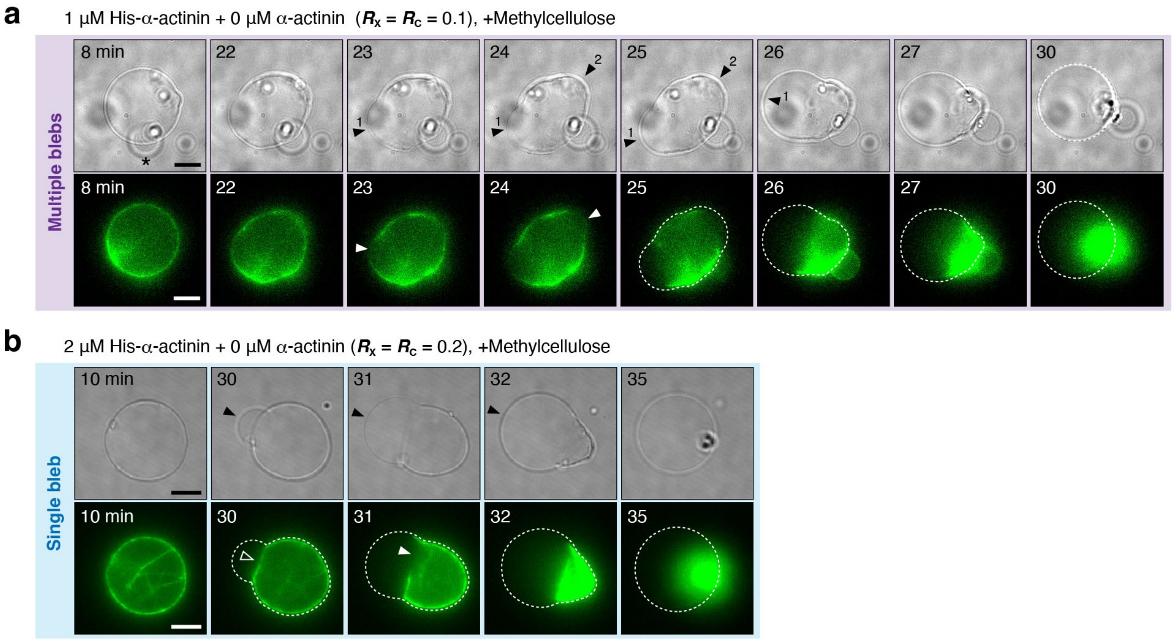

Figure S3. Time-lapse images of liposomes under various conditions.

a, Time-lapse epifluorescence images of the liposome showing double bleb formation (black arrowheads) in the presence of 1 μM His-α-actinin and methylcellulose (**Movie S5**). The asterisk indicates another small liposome attached to the liposome of interest. White arrowheads indicate the rupture points of the actin cortex. **b,** Time-lapse epifluorescence images of the liposome showing single bleb formation (black arrowheads) by the detachment mechanism in the presence of 2 μM His-α-actinin and methylcellulose. After the detachment (white open arrowhead), the cortex was ruptured (white filled arrowhead) (**Movie S8**). This probability was varied between the concentrations of His-α-actinin (C_H) and α-actinin (C_N): 50% (2/4) at [C_H, C_N] = [2,0], 50% (4/8) at [C_H, C_N] = [1,1], 33% (1/3) at [5,0], 10% (1/10) at [2,3], and 9% (1/17) at [1,2]. For all microscopic images, dashed lines indicate the liposome periphery. Scale bars, 10 μm.

bioRxiv preprint doi: https://doi.org/10.1101/2025.05.18.654456; this version posted May 22, 2025. The copyright holder for this preprint (which was not certified by peer review) is the author/funder, who has granted bioRxiv a license to display the preprint in perpetuity. It is made available under aCC-BY 4.0 International license.

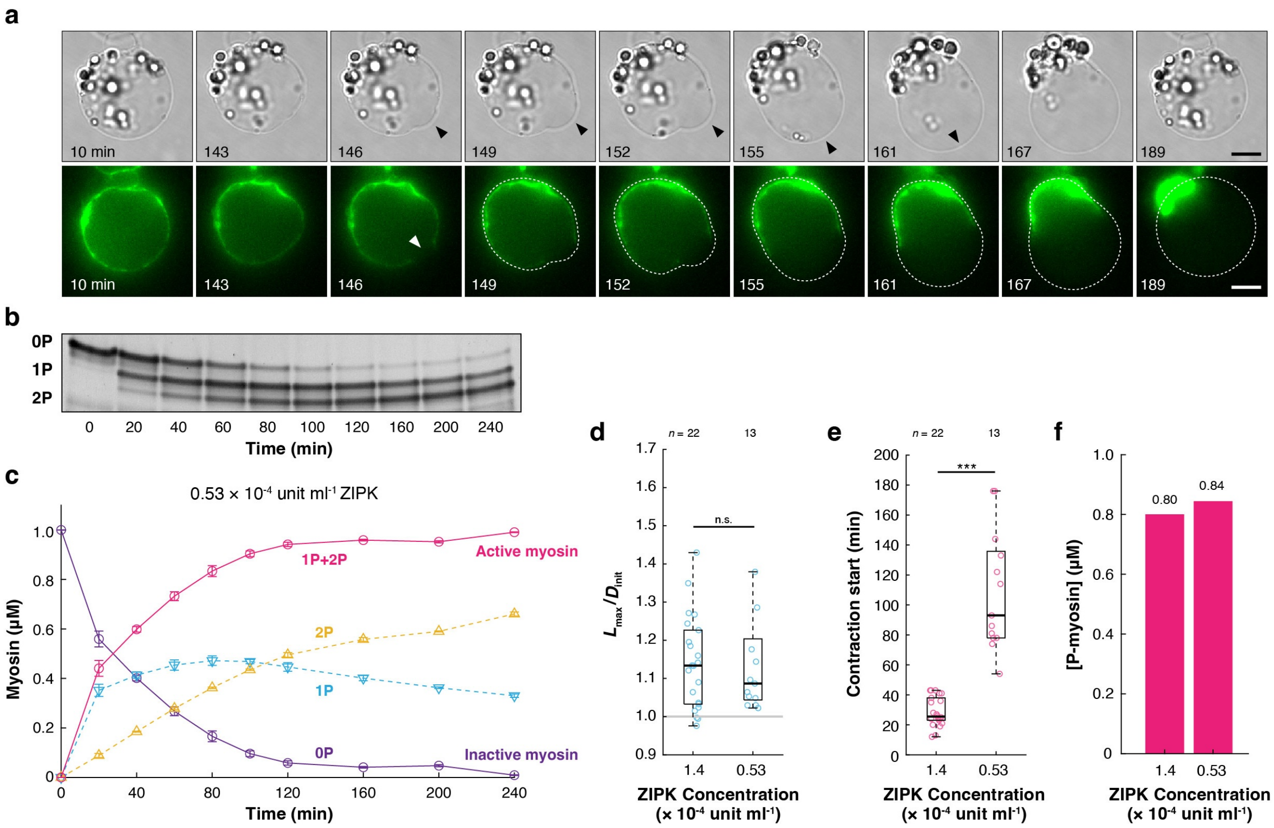

Figure S4. Effects of ZIPK concentration on morphological transition.

a, Time-lapse epifluorescence images of the liposome containing 10 µM actin, 1 µM SMM, 2 µM His-α-actinin, 0.53 × 10^{-4} unit ml^{-1} ZIPK, and 0.2% (w/v) methylcellulose (1,500 cp) (**Movie S10**). Only ZIPK concentration was different from **Fig. 3e,f.** Black arrowheads and a white arrowhead indicate the blebs and the rupture points of the actin cortex, respectively. White dashed lines indicate the liposome periphery. Scale bars, 10 µm. **b,** The image of urea/glycerol PAGE. 1 µM myosin was incubated with 0.53 × 10^{-4} unit ml^{-1} ZIPK in the presence of 1 mM ATP for the indicated times at 25°C. Unphosphorylated (0P), monophosphorylated (1P), and diphosphorylated (2P) myosins were separated by the gel electrophoresis. **c,** Time courses of the phosphorylation reactions of 1 µM SMM in the presence of 0.53 × 10^{-4} unit ml^{-1} ZIPK measured from the band intensities in **c.** Three independent experiments were performed. Error bars indicate the SDs. **d-f,** Comparison of the morphological transition process of liposomes containing two different concentrations of ZIPK. **d,** Magnitude of membrane deformation. **e**, Contraction start time. **f,** Concentration of phosphorylated myosin predicted from the median values in **e,** and the phosphorylation time courses in **c** and **Fig. 1c**. ***: $p < 0.001$, n.s.: $p \geq 0.05$ (Welch's t-test, two-sided).

bioRxiv preprint doi: https://doi.org/10.1101/2025.05.18.654456; this version posted May 22, 2025. The copyright holder for this preprint (which was not certified by peer review) is the author/funder, who has granted bioRxiv a license to display the preprint in perpetuity. It is made available under aCC-BY 4.0 International license.

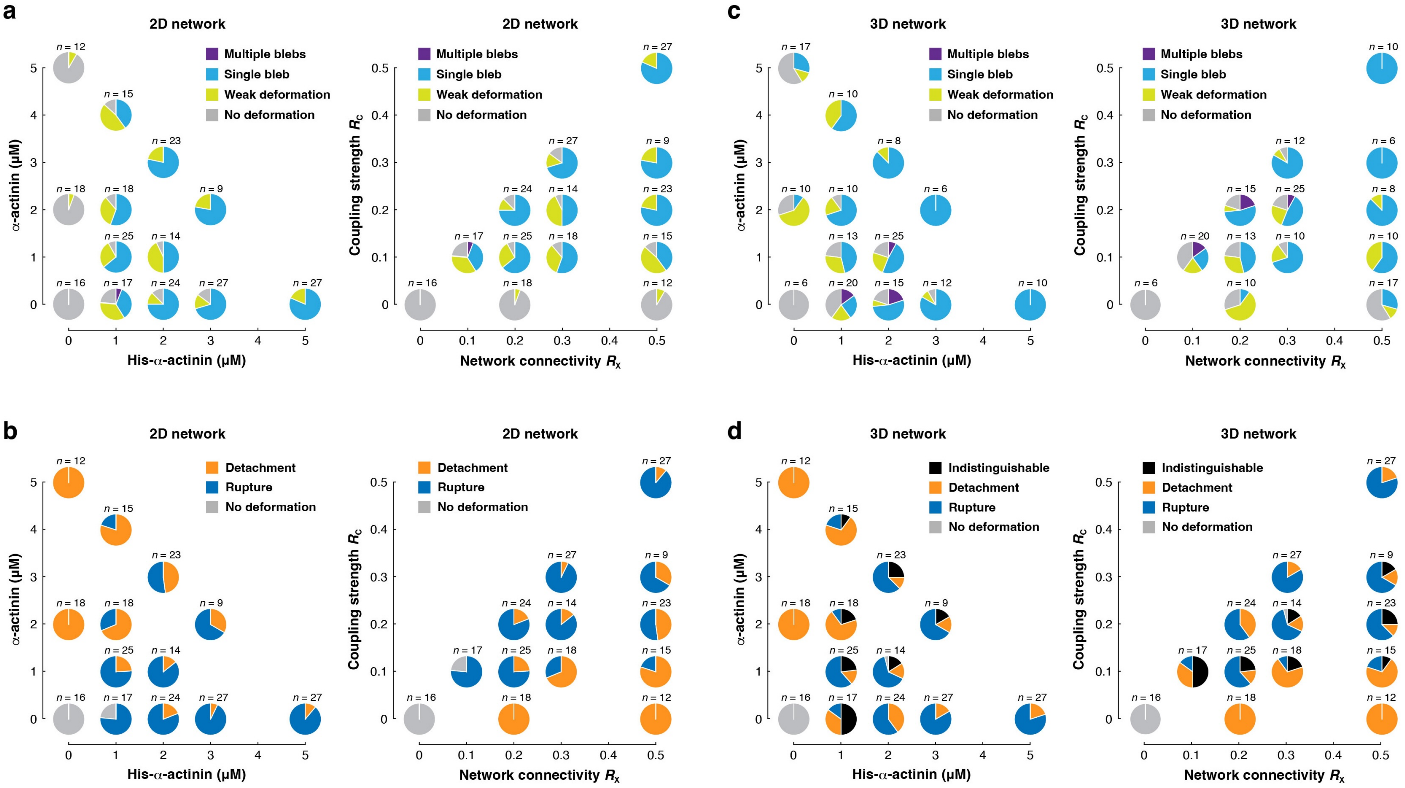

Figure S5. Phase diagram of morphological transition modes and bleb initiation mechanisms in 2D and 3D networks.

a,b, Phase diagrams of (**a**) morphological transition modes and (**b**) bleb initiation mechanisms in the 2D network conditions (in the presence of methylcellulose), (left) plotted using His-α-actinin concentration (C_H) and α-actinin concentration (C_N) as the parameters, and (right) plotted using the network connectivity (R_X) and actin-membrane coupling strength (R_C) as the parameters. **c,d,** The same types of phase diagrams for the 3D network conditions (in the absence of methylcellulose).

bioRxiv preprint doi: https://doi.org/10.1101/2025.05.18.654456; this version posted May 22, 2025. The copyright holder for this preprint (which was not certified by peer review) is the author/funder, who has granted bioRxiv a license to display the preprint in perpetuity. It is made available under aCC-BY 4.0 International license.

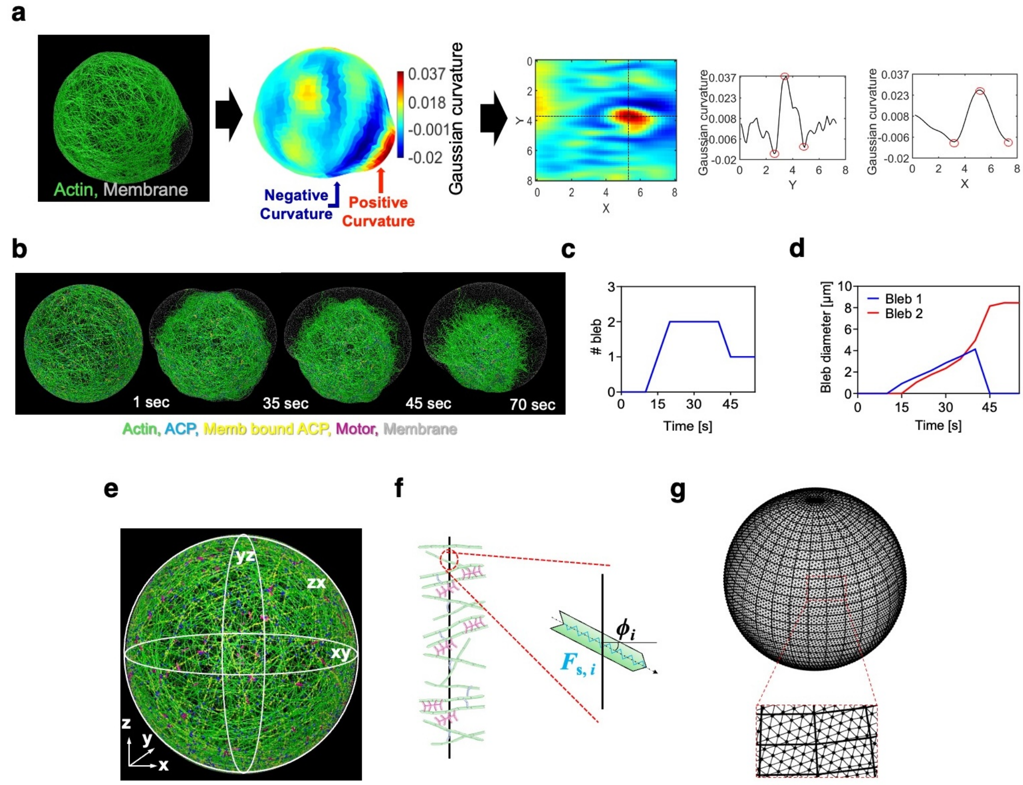

Figure S6. Analysis methods for blebs, global network/membrane tension, and the local density of membrane-bound ACPs.

a, Detection method for blebs. The curvature of the membrane surface was calculated using the discrete mean curvature approximation based on the Voronoi area and uniform triangular edge length. A region with a positive curvature (red) surrounded by negative-curvature regions (blue) was considered a potential location for a bleb. To estimate bleb radius, the apex of the detected bleb was found, and then the mean distance from the apex to the edges of the bleb where the curvature significantly varies was computed. **b-d,** Representative example of the case showing multiple bleb formation. **b,** Time-lapse images. **c,** Quantification of the number of blebs over time by the detection method shown in **a**. **d,** The diameter of two blebs changing over time. **e,f,** Analysis method for global network/membrane tension. (**e**) To assess global tension acting on either the actin network or the membrane, we considered *xy*, *yz*, and *zx* cross-sections that include the instantaneous centroid of the network or the membrane. (**f**) All membrane chains on the triangulated mesh (for membrane tension) or the segments of F-actin, ACPs, and motors (for network tension) crossing each cross-section (indicated by the black line) were identified. The total tension on each cross-section was then determined as the sum of $F_{s,i}$ cos ϕ_i over all chains or segments. **g,** Analysis method for the local density of membrane-bound ACPs. The membrane surface area was discretized into quadrilateral and triangular surface elements using spherical coordinates. Details of all these analyses are described in **Supplementary Note 2,** Sections 9-11.

bioRxiv preprint doi: https://doi.org/10.1101/2025.05.18.654456; this version posted May 22, 2025. The copyright holder for this preprint (which was not certified by peer review) is the author/funder, who has granted bioRxiv a license to display the preprint in perpetuity. It is made available under aCC-BY 4.0 International license.

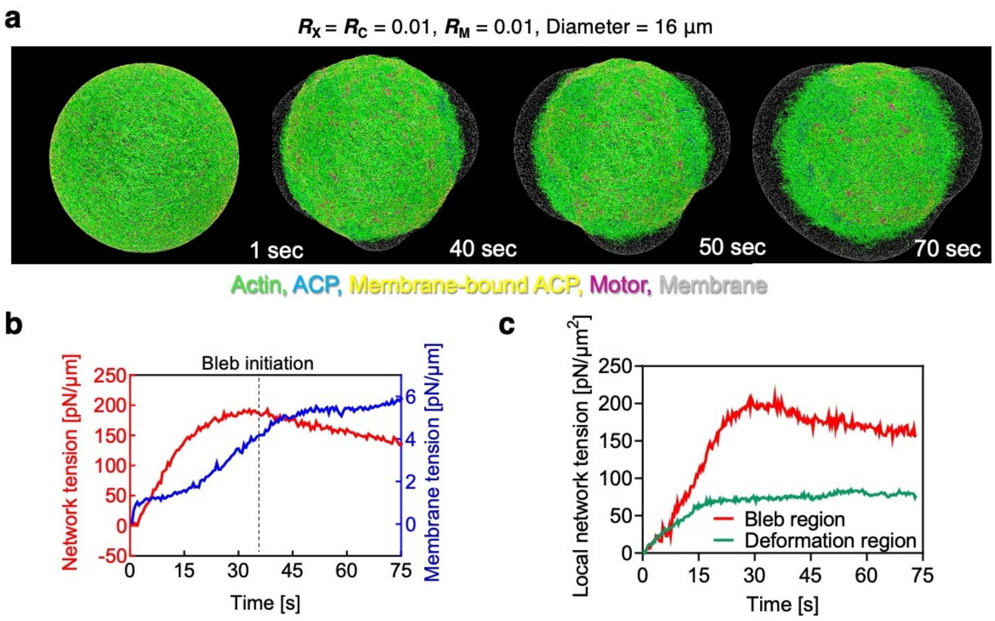

Figure S7. Bleb formation and network/membrane tension observed in a larger vesicle with 16 µm in diameter.

a, Snapshots of the formation, expansion, and mergence of three blebs (**Movie S15**). **b,** Global tension acting on the network (red) and the membrane (blue) over time. The blebs started emerging from ~35 s, so the global network tension decreased from ~35 s. Membrane tension gradually increased from ~35 s. **c,** Comparison of local network tension between the bleb-forming and deformation-only regions until bleb initiation. Local network tension in the bleb-forming region was noticeably higher than that in the deformation-only region.

bioRxiv preprint doi: https://doi.org/10.1101/2025.05.18.654456; this version posted May 22, 2025. The copyright holder for this preprint (which was not certified by peer review) is the author/funder, who has granted bioRxiv a license to display the preprint in perpetuity. It is made available under aCC-BY 4.0 International license.

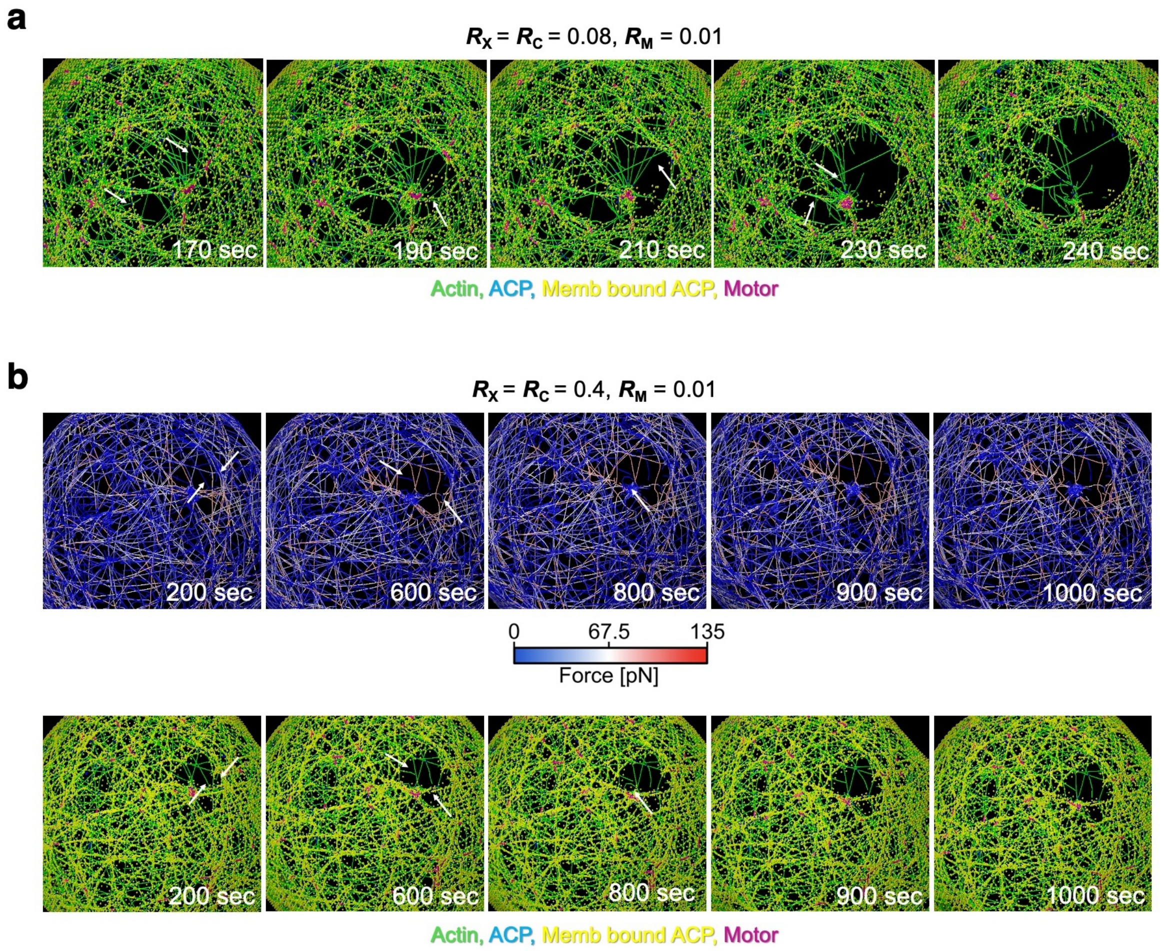

Figure S8. Time evolution of a network rupture induced by F-actin severing.
a, Intermediate network connectivity and actin-membrane coupling strength ($R_X = R_C = 0.08$). Successive severing events of F-actin indicated by arrows led to the initial formation and expansion of a rupture on a network. **b,** High network connectivity and actin-membrane coupling strength ($R_X = R_C = 0.4$). Several severing events still occurred, but a network rupture was not followed by significant expansion.

bioRxiv preprint doi: https://doi.org/10.1101/2025.05.18.654456; this version posted May 22, 2025. The copyright holder for this preprint (which was not certified by peer review) is the author/funder, who has granted bioRxiv a license to display the preprint in perpetuity. It is made available under aCC-BY 4.0 International license.

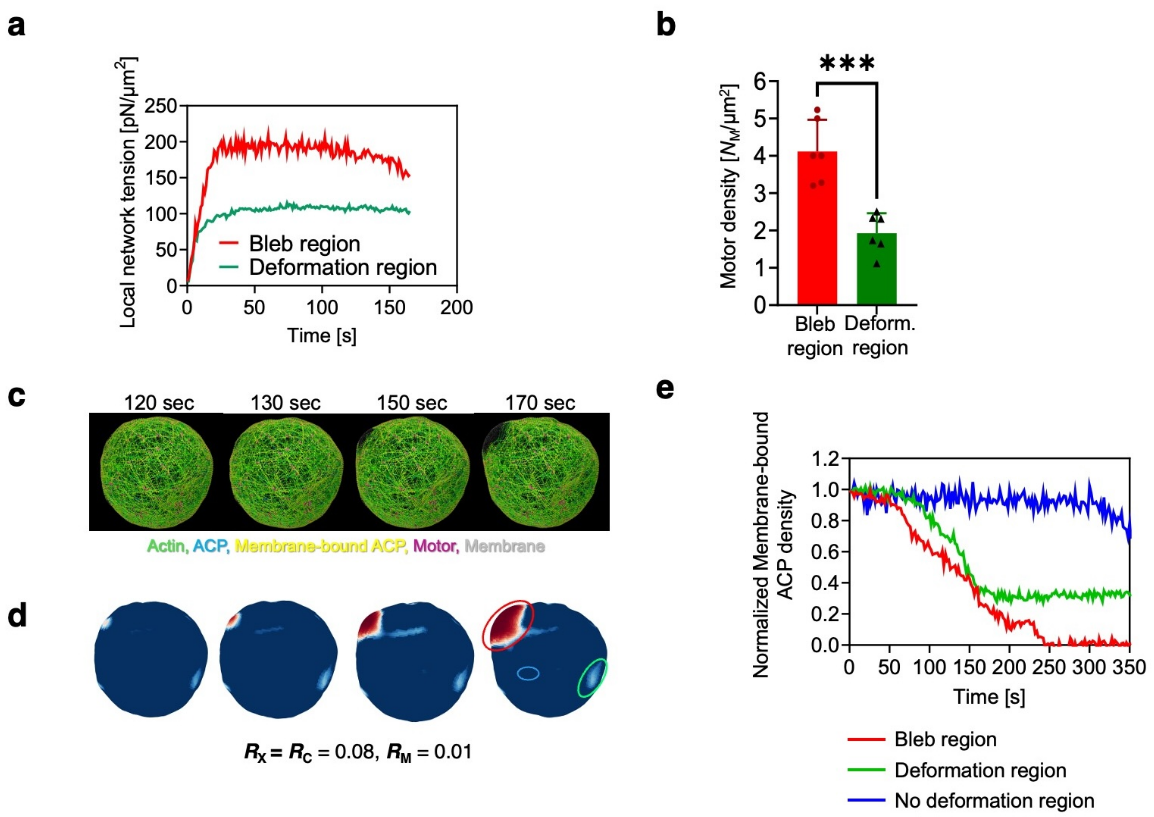

Figure S9. Analysis of local tension, motor density, and actin-membrane coupling. **a,** Comparison of local network tension between the bleb-forming and deformation-only regions developing until bleb formation (The bleb was detected at t = ~150 s in this example). These measurements were performed using the same vesicle. Local network tension measured near the bleb-forming region was substantially higher than that measured in the deformation-only region. **b,** Local motor density measured in the two regions shown in **a**. The density shows a substantial difference, implying that locally higher actomyosin contractility led to a network rupture. ***: $p <$ 0.001 (Welch's t-test, two-sided). **c,** Snapshots of the formation of a single bleb at four different time points. **d,** Quantification of the local density of membrane-bound ACPs in the same vesicle at the same time points as **c**. Not all regions with lower density evolved to a bleb. **e,** The local density of membrane-bound ACPs developing over time in bleb-forming (red line), deformation-only (green line), and no-deformation (blue line) regions, as shown in **d** with red, green, and blue circles, respectively. The local density continuously decreased to nearly zero only in the bleb-forming region. The deformation-only region also showed a decrease in the local density, but the local density stopped decreasing and reached a plateau after substantially decreasing, and the network rupture did not expand significantly. The no-deformation region did not show a noticeable decrease in the local density.

bioRxiv preprint doi: https://doi.org/10.1101/2025.05.18.654456; this version posted May 22, 2025. The copyright holder for this preprint (which was not certified by peer review) is the author/funder, who has granted bioRxiv a license to display the preprint in perpetuity. It is made available under aCC-BY 4.0 International license.

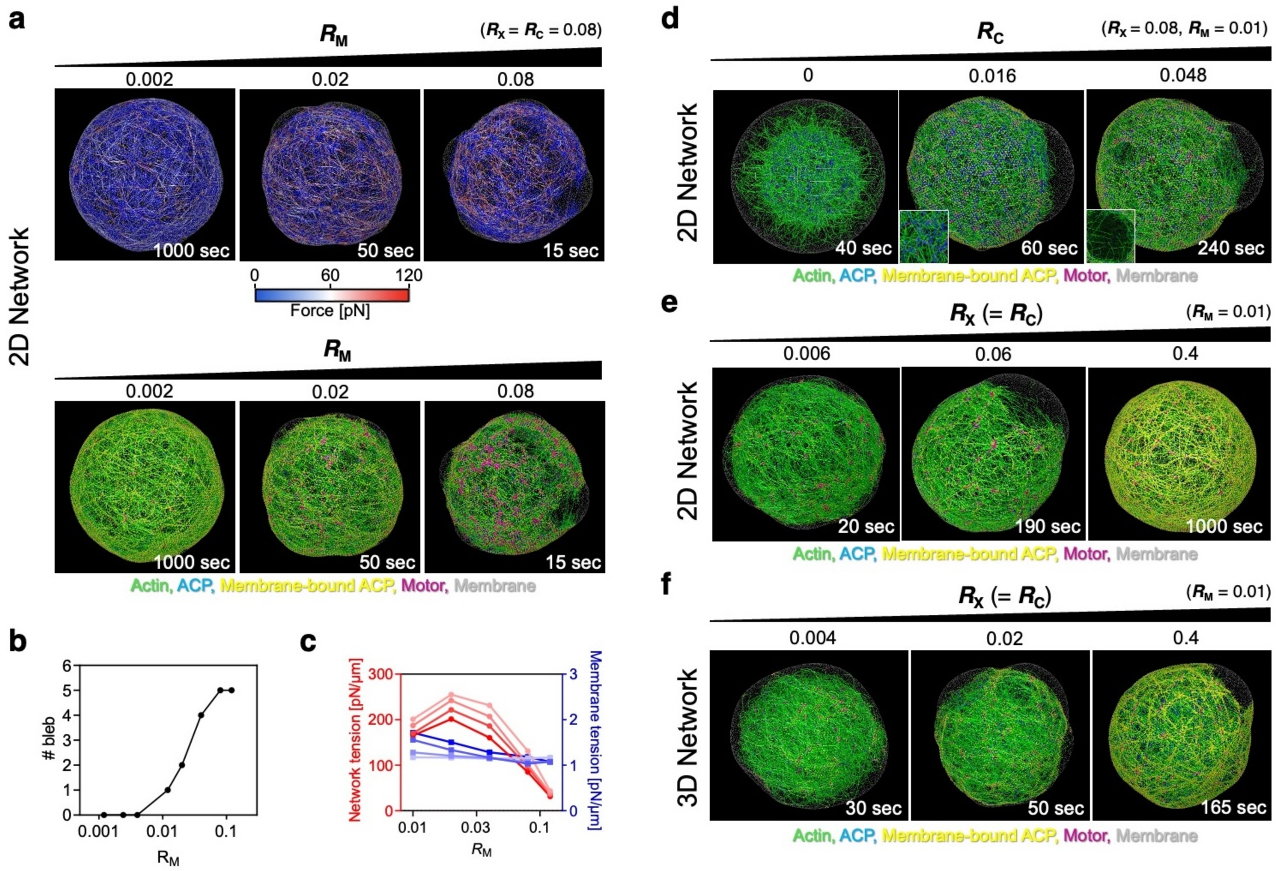

Figure S10. Morphological transitions and bleb formation under different conditions.

a-c, Effects of the motor density, R_M (R_X = R_C = 0.08). **a,** Representative snapshots of the vesicles with different R_M. (top) Force distribution. (bottom) F-actin, ACPs, motors, and a membrane shown by different colors. ACPs are shown in two different colors, depending on whether or not they are coupled to the membrane. **b,** Number of blebs as a function of R_M. **c,** Network and membrane tension with a variation in R_M. Higher R_M led to decreased network tension because of impaired connectivity due to several F-actin severing events, whereas membrane tension hardly varied. **d-f,** Snapshots showing the same structures as those shown in **Fig. 7a, e,** and **i**, respectively. Instead of forces, these snapshots show F-actin, ACPs, motors, and a membrane via different colors. ACPs are shown in two different colors, depending on whether or not they are coupled to the membrane.

bioRxiv preprint doi: https://doi.org/10.1101/2025.05.18.654456; this version posted May 22, 2025. The copyright holder for this preprint (which was not certified by peer review) is the author/funder, who has granted bioRxiv a license to display the preprint in perpetuity. It is made available under aCC-BY 4.0 International license.

SUPPLEMENTARY NOTE 1

In the absence of the hydrostatic pressure ($\Delta P = 0$), the actin-bound His-α-actinin density $\rho_{\mathrm{H}}^{\mathrm{b}}$ is governed by the kinetic equation:

$$\frac{\mathrm{d}\rho_{\mathrm{H}}^{\mathrm{b}}}{\mathrm{d}t} = k_{\mathrm{on}}\left(\rho_{\mathrm{H}} - \rho_{\mathrm{H}}^{\mathrm{b}}\right)\left(\rho_{\mathrm{A}} - \rho_{\mathrm{H}}^{\mathrm{b}} - \rho_{\mathrm{N}}^{\mathrm{b}}\right) - k_{\mathrm{off}}\rho_{\mathrm{H}}^{\mathrm{b}}, \qquad \text{(S1)}$$

where ρ_{H}, $\rho_{\mathrm{H}}^{\mathrm{b}}$, $\rho_{\mathrm{N}}^{\mathrm{b}}$, and ρ_{A} indicate the surface densities of all His-α-actinin, His-α-actinin bound on F-actin, α-actinin bound on F-actin, and all F-actin beneath the membrane, respectively. k_{on} and k_{off} are the rate constants for binding and unbinding between His-α-actinin and F-actin, respectively. Considering that ΔP tends to separate the membrane from the actin cortex, and assuming that each His-α-actinin-F-actin bond is equally stressed, the force per bond, f, becomes $f = \Delta P/\rho_{\mathrm{H}}^{\mathrm{b}}$. Using Bell's kinetics model[1], the kinetic equation (**Eq. S1**) is replaced by

$$\frac{\mathrm{d}\rho_{\mathrm{H}}^{\mathrm{b}}}{\mathrm{d}t} = k_{\mathrm{on}}\left(\rho_{\mathrm{H}} - \rho_{\mathrm{H}}^{\mathrm{b}}\right)\left(\rho_{\mathrm{A}} - \rho_{\mathrm{H}}^{\mathrm{b}} - \rho_{\mathrm{N}}^{\mathrm{b}}\right) - k_{\mathrm{off}}^{0}\rho_{\mathrm{H}}^{\mathrm{b}} \exp\left(\frac{r_0\Delta P}{k_{\mathrm{B}}T\rho_{\mathrm{H}}^{\mathrm{b}}}\right), \qquad \text{(S2)}$$

where k_{off}^{0} is the zero-force unbinding rate constant, and r_0 is a characteristic distance to overcome the energetic barrier for bond separation[1-3]. If it is assumed that F-actin near the membrane retains many free binding sites for α-actinin ($\rho_{\mathrm{A}} \gg \rho_{\mathrm{H}}^{\mathrm{b}} + \rho_{\mathrm{N}}^{\mathrm{b}}$), **Eq. S2** can be replaced by

$$\begin{aligned}\frac{\mathrm{d}\rho_{\mathrm{H}}^{\mathrm{b}}}{\mathrm{d}t} &= k_{\mathrm{on}}\left(\rho_{\mathrm{H}} - \rho_{\mathrm{H}}^{\mathrm{b}}\right)\rho_{\mathrm{A}} - k_{\mathrm{off}}^{0}\rho_{\mathrm{H}}^{\mathrm{b}} \exp\left(\frac{r_0\Delta P}{k_{\mathrm{B}}T\rho_{\mathrm{H}}^{\mathrm{b}}}\right) \\ &= g\left(\rho_{\mathrm{H}}^{\mathrm{b}}\right) - h\left(\rho_{\mathrm{H}}^{\mathrm{b}}, \Delta P\right), \end{aligned} \qquad \text{(S3)}$$

where $g\left(\rho_{\mathrm{H}}^{\mathrm{b}}\right) \equiv k_{\mathrm{on}}\left(\rho_{\mathrm{H}} - \rho_{\mathrm{H}}^{\mathrm{b}}\right)\rho_{\mathrm{A}}$ and $h\left(\rho_{\mathrm{H}}^{\mathrm{b}}, \Delta P\right) \equiv k_{\mathrm{off}}^{0}\rho_{\mathrm{H}}^{\mathrm{b}} \exp\left(r_0\Delta P/k_{\mathrm{B}}T\rho_{\mathrm{H}}^{\mathrm{b}}\right)$ are the binding and unbinding rates, respectively. At the initial condition ($t = 0$), $\Delta P \simeq 0$ because forces generated by myosin are negligibly small. If it is assumed that the system is initially at an equilibrium ($\mathrm{d}\rho_{\mathrm{H}}^{\mathrm{b}}/\mathrm{d}t = 0$), **Eq. S3** becomes

$$g\left(\rho_{\mathrm{H}}^{\mathrm{b}}(0)\right) = h\left(\rho_{\mathrm{H}}^{\mathrm{b}}(0), 0\right). \qquad \text{(S4)}$$

Therefore, we obtain

$$\rho_{\mathrm{H}}^{\mathrm{b}}(0) = \frac{\rho_{\mathrm{A}}\rho_{\mathrm{H}}}{\rho_{\mathrm{A}} + K_{\mathrm{m}}^{2\mathrm{D}}}, \qquad \text{(S5)}$$

where $K_{\mathrm{m}}^{2\mathrm{D}} = k_{\mathrm{off}}^{0}/k_{\mathrm{on}}$ is the dissociation constant of His-α-actinin from F-actin in a 2D network. Subsequently, myosin is gradually phosphorylated and begins to generate contractile forces on the cortical actin network, leading to an increase in ΔP. A small increase in ΔP

bioRxiv preprint doi: https://doi.org/10.1101/2025.05.18.654456; this version posted May 22, 2025. The copyright holder for this preprint (which was not certified by peer review) is the author/funder, who has granted bioRxiv a license to display the preprint in perpetuity. It is made available under aCC-BY 4.0 International license.

slightly elevates the unbinding rate, $h(\rho_{\mathrm{H}}^{\mathrm{b}}, \Delta P)$, leading to a slight decrease in $\rho_{\mathrm{H}}^{\mathrm{b}}$, after which the system is expected to reach a new equilibrium ($\mathrm{d}\rho_{\mathrm{H}}^{\mathrm{b}}/\mathrm{d}t = 0$). By contrast, when large ΔP is applied, the unbinding rate, $h(\rho_{\mathrm{H}}^{\mathrm{b}}, \Delta P)$, becomes much larger than the binding rate, $g(\rho_{\mathrm{H}}^{\mathrm{b}})$, causing $\rho_{\mathrm{H}}^{\mathrm{b}}$ to rapidly approach zero. There will thus exist the critical pressure, ΔP^{*}, that is sufficient to detach all His-α-actinin from actin filaments.

To evaluate the critical pressure, ΔP^{*}, we consider the balance between binding and unbinding rates, $g(\rho_{\mathrm{H}}^{\mathrm{b}})$ and $h(\rho_{\mathrm{H}}^{\mathrm{b}}, \Delta P)$, which are plotted in **Fig. SN1** as functions of $\rho_{\mathrm{H}}^{\mathrm{b}}$. The unbinding rate term (blue lines) contains $\Delta P(t)$ as a parameter. At the initial state ($t = 0$) when myosin-generated force is small enough to be negligible as described above: $\Delta P(0) \simeq 0$, both terms are linear and intersect at a single point (**Fig. SN1,** cross), which gives a stable equilibrium ($\mathrm{d}\rho_{\mathrm{H}}^{\mathrm{b}}/\mathrm{d}t = 0$). At $t > 0$, $\Delta P(t)$ gradually increases as myosin phosphorylation proceeds, rendering the unbinding rate term nonlinear. Consequently, the two terms have two intersections: a stable fixed point (**Fig. SN1,** filled circle) and an unstable fixed point (**Fig. SN1,** open circle). Since $\rho_{\mathrm{H}}^{\mathrm{b}}(t)$ at the stable fixed point is smaller than $\rho_{\mathrm{H}}^{\mathrm{b}}(0)$ and $g(\rho_{\mathrm{H}}^{\mathrm{b}}) - h(\rho_{\mathrm{H}}^{\mathrm{b}}, \Delta P)$ is negative between $\rho_{\mathrm{H}}^{\mathrm{b}}(t)$ and $\rho_{\mathrm{H}}^{\mathrm{b}}(0)$, $\rho_{\mathrm{H}}^{\mathrm{b}}(t)$ reaches the stable fixed point for each t (> 0). A further increase in $\Delta P(t)$ shifts the unbinding rate curve upward, decreasing $\rho_{\mathrm{H}}^{\mathrm{b}}(t)$ along the red line. When $\Delta P(t)$ reaches ΔP^{*}, the two terms are tangent, and the stable and unstable fixed points merge at $\rho_{\mathrm{H}}^{\mathrm{b}} = \rho_{\mathrm{H}}^{\mathrm{b}*}$. For $\Delta P(t) > \Delta P^{*}$, no stable fixed point exists, and $g(\rho_{\mathrm{H}}^{\mathrm{b}}) - h(\rho_{\mathrm{H}}^{\mathrm{b}}, \Delta P)$ is negative for all $\rho_{\mathrm{H}}^{\mathrm{b}}$. Hence, $\rho_{\mathrm{H}}^{\mathrm{b}}(t)$ decreases monotonically to zero, eventually leading to the complete detachment of actin filaments from the membrane. Therefore, ΔP^{*} is the critical pressure that initiates "Detachment".

Using the facts that the binding and unbinding rates are equal and their slopes are also equal at the point of tangency, $\rho_{\mathrm{H}}^{\mathrm{b}*}$ and ΔP^{*} satisfy the following equations,

$$g(\rho_{\mathrm{H}}^{\mathrm{b}*}) = h(\rho_{\mathrm{H}}^{\mathrm{b}*}, \Delta P^{*}) \,, \tag{S6}$$

$$\left.\frac{\mathrm{d}g(\rho_{\mathrm{H}}^{\mathrm{b}})}{\mathrm{d}\rho_{\mathrm{H}}^{\mathrm{b}}}\right|_{\rho_{\mathrm{H}}^{\mathrm{b}} = \rho_{\mathrm{H}}^{\mathrm{b}*}} = \left.\frac{\mathrm{d}h(\rho_{\mathrm{H}}^{\mathrm{b}}, \Delta P^{*})}{\mathrm{d}\rho_{\mathrm{H}}^{\mathrm{b}}}\right|_{\rho_{\mathrm{H}}^{\mathrm{b}} = \rho_{\mathrm{H}}^{\mathrm{b}*}} \,, \tag{S7}$$

which lead to

$$(\rho_{\mathrm{H}} - \rho_{\mathrm{H}}^{\mathrm{b}*})\rho_{\mathrm{A}} = K_{\mathrm{m}}^{\mathrm{2D}} \rho_{\mathrm{H}}^{\mathrm{b}*} \exp\left(\frac{r_0 \Delta P^{*}}{k_{\mathrm{B}} T \rho_{\mathrm{H}}^{\mathrm{b}*}}\right) \,, \tag{S8}$$

$$-\rho_{\mathrm{A}} = K_{\mathrm{m}}^{\mathrm{2D}} \exp\left(\frac{r_0 \Delta P^{*}}{k_{\mathrm{B}} T \rho_{\mathrm{H}}^{\mathrm{b}*}}\right) - K_{\mathrm{m}}^{\mathrm{2D}} \frac{r_0 \Delta P^{*}}{k_{\mathrm{B}} T \rho_{\mathrm{H}}^{\mathrm{b}*}} \exp\left(\frac{r_0 \Delta P^{*}}{k_{\mathrm{B}} T \rho_{\mathrm{H}}^{\mathrm{b}*}}\right) . \tag{S9}$$

bioRxiv preprint doi: https://doi.org/10.1101/2025.05.18.654456; this version posted May 22, 2025. The copyright holder for this preprint (which was not certified by peer review) is the author/funder, who has granted bioRxiv a license to display the preprint in perpetuity. It is made available under aCC-BY 4.0 International license.

By combining **Eq. S8** and **Eq. S9** and eliminating $\rho_{\mathrm{H}}^{\mathrm{b}*}$, we obtain the following equation:

$$\alpha^* \exp(1+\alpha^*) = \frac{\rho_{\mathrm{A}}}{K_{\mathrm{m}}^{\mathrm{2D}}}, \tag{S10}$$

where

$$\alpha^* = \frac{r_0}{k_{\mathrm{B}} T \rho_{\mathrm{H}}} \Delta P^* . \tag{S11}$$

As shown in **Fig. SN2,**

$$\alpha^* \simeq 0.7 \ln\left(\frac{\rho_{\mathrm{A}}}{K_{\mathrm{m}}^{\mathrm{2D}}}\right) \tag{S12}$$

for an extensive range of $\rho_{\mathrm{A}}/K_{\mathrm{m}}^{\mathrm{2D}}$. Therefore, by substituting **Eq. S12** to **Eq. S11,** we obtain the critical pressure for “Detachment”:

$$\Delta P^* \simeq \frac{0.7\, k_{\mathrm{B}} T \rho_{\mathrm{H}}}{r_0} \ln\left(\frac{\rho_{\mathrm{A}}}{K_{\mathrm{m}}^{\mathrm{2D}}}\right). \tag{S13}$$

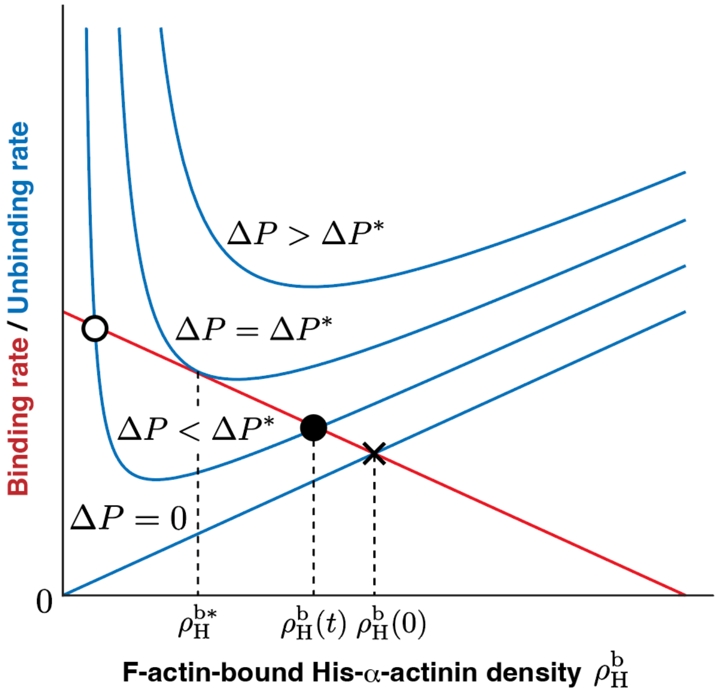

Figure SN1. Relationship between the binding and unbinding rates (the first and second terms on the right-hand side of **Eq. S3,** respectively) as a function of F-actin-bound His-α-actinin density, $\rho_{\mathrm{H}}^{\mathrm{b}}$. At $t = 0$, the two rates are balanced at a single intersection point (cross). As t increases and $\Delta P(t)$ rises, a stable fixed point (filled circle) and an unstable fixed point (open circle) gradually approach each other and merge at the critical pressure, ΔP^*.

bioRxiv preprint doi: https://doi.org/10.1101/2025.05.18.654456; this version posted May 22, 2025. The copyright holder for this preprint (which was not certified by peer review) is the author/funder, who has granted bioRxiv a license to display the preprint in perpetuity. It is made available under aCC-BY 4.0 International license.

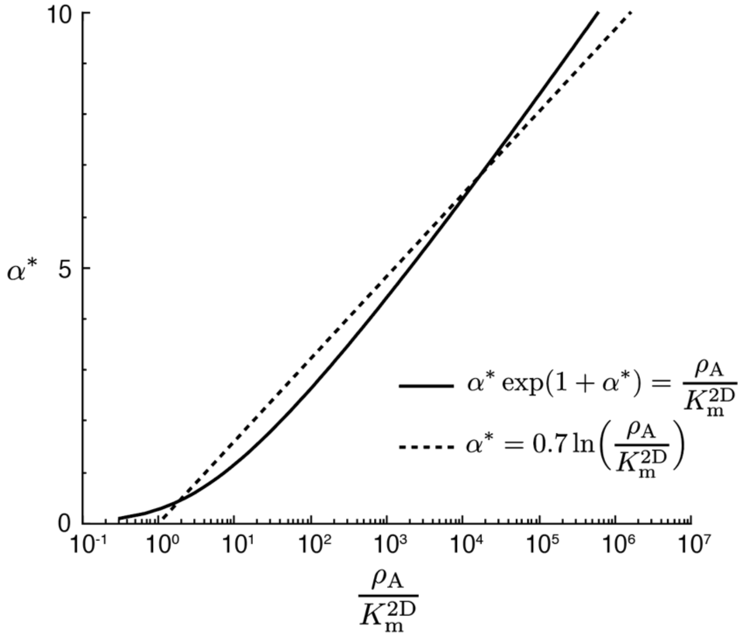

Figure SN2. Comparison between **Eq. S10** (solid line) and **Eq. S12** (dashed line).

REFERENCES

1. Bell, G. Models for the specific adhesion of cells to cells. *Science* **200**, 618–627 (1978).
2. Miyata, H., Yasuda, R. & Kinosita, K. Strength and lifetime of the bond between actin and skeletal muscle α-actinin studied with an optical trapping technique. *Biochim. Biophys. Acta* **1290**, 83–88 (1996).
3. Ferrer, J. M., Lee, H., Chen, J., Pelz, B., Nakamura, F., Kamm, R. D. & Lang, M. J. Measuring molecular rupture forces between single actin filaments and actin-binding proteins. *Proc. Natl Acad. Sci. USA* **105,** 9221–9226 (2008).

bioRxiv preprint doi: https://doi.org/10.1101/2025.05.18.654456; this version posted May 22, 2025. The copyright holder for this preprint (which was not certified by peer review) is the author/funder, who has granted bioRxiv a license to display the preprint in perpetuity. It is made available under aCC-BY 4.0 International license.

SUPPLEMENTARY NOTE 2

1. Brownian dynamics simulations via the Langevin equation.

In the agent-based model, elements are defined by points (i.e., nodes), and cylindrical segments exist between the points. The displacements of points are governed by the Langevin equation with inertia neglected:

$$\mathbf{F}_i - \zeta_i \frac{\mathrm{d}\mathbf{r}_i}{\mathrm{d}t} + \mathbf{F}_i^{\mathrm{T}} = 0 \tag{S1}$$

where $\mathbf{r}_i$ is the position of the ith point, ζ_i is a drag coefficient, t is time, $\mathbf{F}_i$ is a deterministic force, and $\mathbf{F}_i^{\mathrm{T}}$ is a stochastic force. The magnitude of $\mathbf{F}_i^{\mathrm{T}}$ is determined by the fluctuation-dissipation theorem:

$$\left\langle \mathbf{F}_i^{\mathrm{T}}(t)\mathbf{F}_j^{\mathrm{T}}(t) \right\rangle = \frac{2k_{\mathrm{B}}T\zeta_i\delta_{ij}}{\Delta t}\boldsymbol{\delta} \tag{S2}$$

where δ_{ij} is the Kronecker delta, $\boldsymbol{\delta}$ is a second-order tensor, and Δt is a time step. In most simulations, Δt is 1.15×10^{-4} s.

The drag coefficients of points are computed using an approximated form for a cylindrical object[1]:

$$\zeta_i = 3\pi\mu d_i \frac{3+2l_i/d_i}{5} \tag{S3}$$

where μ is the viscosity of a medium, and l_i and d_i are the length and diameter of a cylindrical segment connected to the point, respectively. The positions of points are updated at each time step using the Euler integration scheme:

$$\mathbf{r}_i(t+\Delta t) = \mathbf{r}_i(t) + \frac{\mathrm{d}\mathbf{r}_i}{\mathrm{d}t}\Delta t = \mathbf{r}_i(t) + \frac{1}{\zeta_i}\left(\mathbf{F}_i + \mathbf{F}_i^{\mathrm{T}}\right)\Delta t \tag{S4}$$

Three types of deterministic forces maintain lengths, distances, and angles involved with components close to their equilibrium values. Extensional forces maintain the equilibrium lengths of cylindrical segments or chains, bending forces maintain angles formed by interconnected segments or dihedral angles, and repulsive forces represent volume-exclusion effects acting between overlapping components. The extensional and bending forces originate from harmonic potentials:

$$U_{\mathrm{s}} = \frac{1}{2}\kappa_{\mathrm{s}}(l-l_0)^2 \tag{S5}$$

$$U_{\mathrm{b}} = \frac{1}{2}\kappa_{\mathrm{b}}\left(\theta-\theta_0\right)^2 \tag{S6}$$

bioRxiv preprint doi: https://doi.org/10.1101/2025.05.18.654456; this version posted May 22, 2025. The copyright holder for this preprint (which was not certified by peer review) is the author/funder, who has granted bioRxiv a license to display the preprint in perpetuity. It is made available under aCC-BY 4.0 International license.

where κ_s is extensional stiffness, l and l_0 are instantaneous and equilibrium lengths, κ_b is bending stiffness, and θ and θ_0 are instantaneous and equilibrium angles. All details of this model are explained below.

2. Simplification and mechanics of cytoskeletal components.

F-actin is simplified into serially connected cylindrical segments. Each segment has polarity defined by barbed and pointed ends. Extensional stiffness of F-actin ($\kappa_{s,A}$) maintains the length of actin segments close to its equilibrium ($l_{0,A}$ = 140 nm). Bending stiffness of F-actin ($\kappa_{b,A}$) maintains an angle formed by two adjacent actin segments close to its equilibrium ($\theta_{0,A}$ = 0 rad). The persistence length of F-actin (l_p) calculated from $\kappa_{b,A}$ and k_BT corresponds to ~9 μm (ref. 2).

Actin crosslinking proteins (ACPs) consist of two cylindrical segments connected at their center point. Extensional stiffness of ACPs maintains the length of each ACP segment close to its equilibrium ($l_{0,ACP}$ = 23.5 nm). Bending stiffness of ACPs ($\kappa_{b,ACP}$) maintains an angle formed by two ACP segments at the center point close its equilibrium ($\theta_{0,ACP}$ = 0 rad). The geometry of ACPs is analogous to α-actinin[3].

Each motor has a backbone structure with 7 segments and 8 points. On each point, two motor arms are connected, so the total number of motor arms is 16 (N_{arm} = 16). Each arm represents the kinetics and force-velocity relationship of the ensemble consisting of 4 myosin heads (N_h = 4). Thus, the total number of myosin heads represented by each motor is 64, which is not far from ~56 myosin heads in a single non-muscle myosin mini-filament[4]. The equilibrium length of each motor backbone segment ($l_{0,M1}$ = 42 nm) is regulated by extensional stiffness ($\kappa_{s,M1}$). Thus, the equilibrium length of a whole motor backbone is 294 nm, nearly equal to the length of non-muscle myosin mini-filaments[5]. An equilibrium angle formed by adjacent backbone segments ($\theta_{0,M}$ = 0 rad) is regulated by bending stiffness ($\kappa_{b,M}$). The extension of each motor arm is regulated by the two-spring model with the stiffnesses of transverse ($\kappa_{s,M2}$) and longitudinal ($\kappa_{s,M3}$) springs. The transverse spring regulates an equilibrium distance ($l_{0,M2}$ = 13.5 nm) between the backbone point where the motor arm is connected and an actin segment where the other end of the motor arm is bound, and the longitudinal spring maintains a right angle between the motor arm and the actin segment ($l_{0,M3}$ = 0 nm).

Forces exerted on actin segments by bound ACPs and motors are distributed to the barbed and pointed ends of the actin segments as described in our previous work[6]. In the

bioRxiv preprint doi: https://doi.org/10.1101/2025.05.18.654456; this version posted May 22, 2025. The copyright holder for this preprint (which was not certified by peer review) is the author/funder, who has granted bioRxiv a license to display the preprint in perpetuity. It is made available under aCC-BY 4.0 International license.

network, repulsive forces are calculated only between actin filaments (among cytoskeletal components), following a harmonic potential[6]:

$$U_{\mathrm{r,A}} = \begin{cases} \frac{1}{2}\kappa_{\mathrm{r,A}}\left(r_{12} - d_{\mathrm{A}}\right)^2 & \text{if} \quad r_{12} < d_{\mathrm{A}} \\ 0 & \text{if} \quad r_{12} \geq d_{\mathrm{A}} \end{cases} \tag{S7}$$

where $\kappa_{\mathrm{r,A}}$ is the strength of the repulsive force, r_{12} is a minimum distance between two neighboring actin segments, and d_{A} is the diameter of the actin segment.

3. Simplification and mechanics of the membrane.

A cell membrane is simplified into a triangulated mesh with area and volume conservation. Extensional stiffness of the membrane ($\kappa_{\mathrm{s,MB}}$) governs the equilibrium length of chains ($l_{0,\mathrm{MB}}$ = ~70 nm) constituting the mesh. Bending stiffness of the membrane ($\kappa_{\mathrm{b,MB}}$) regulates dihedral angles formed by adjacent triangular elements on the mesh close to their equilibrium value ($\theta_{0,\mathrm{MB}}$ = 0 rad). The area of each triangular element and the entire volume within the membrane are conserved following harmonic potentials:

$$U_{\mathrm{V,MB}} = \frac{1}{2}\kappa_{\mathrm{V,MB}}\left(V_{\mathrm{MB}} - V_{0,\mathrm{MB}}\right)^2 \tag{S8}$$

$$U_{\mathrm{A,MB}} = \frac{1}{2}\kappa_{\mathrm{A,MB}}\left(A_{\mathrm{MB}} - A_{0,\mathrm{MB}}\right)^2 \tag{S9}$$

where $\kappa_{\mathrm{V,MB}}$ and $\kappa_{\mathrm{A,MB}}$ are the strengths of volume and area conservation, V_{MB} is volume, A_{MB} is area, and the subscript 0 denotes the equilibrium value.

Repulsive forces are applied between triangular elements on the mesh with strength, $\kappa_{\mathrm{r,MB}}$, to prevent them from crossing each other. The repulsive forces originate from the following potential:

$$U_{\mathrm{r,MB}} = \begin{cases} \kappa_{\mathrm{r,MB}}\left(r_{12} - r_{0,\mathrm{r}}\right)^2 \left[\frac{1}{2} + \frac{1}{3}\left(1 - \frac{r_{12}}{r_{0,\mathrm{r}}}\right)^4\right] & \text{if} \quad r_{12} < r_{0,\mathrm{r}} \\ 0 & \text{if} \quad r_{12} \geq r_{0,\mathrm{r}} \end{cases} \tag{S10}$$

where $\kappa_{\mathrm{r,MB}}$ is the strength of the repulsive force, and $r_{0,\mathrm{r}}$ is a distance below which the repulsive force starts acting. In this case, $r_{0,\mathrm{r}}$ is equal to the thickness of the membrane mesh ($r_{0,\mathrm{r}} = d_{\mathrm{MB}}$). Note that this repulsive force increases more rapidly as the two membrane elements get closer to each other.

bioRxiv preprint doi: https://doi.org/10.1101/2025.05.18.654456; this version posted May 22, 2025. The copyright holder for this preprint (which was not certified by peer review) is the author/funder, who has granted bioRxiv a license to display the preprint in perpetuity. It is made available under aCC-BY 4.0 International license.

4. Actin dynamics.

The formation of F-actin is initiated from a nucleation event occurring at a constant rate, $k_{n,A}$, with the appearance of one cylindrical segment in a random position. The polymerization of F-actin is simulated by adding cylindrical segments at the barbed end of existing filaments at a constant rate, $k_{+,A}$. The nucleation and polymerization events are skipped if any part of the new segment is located outside a designated space for network assembly. The depolymerization of F-actin does not take place. The severing of F-actin occurs in a deterministic manner; if a tensile force acting on an actin segment becomes greater than F_{sev} = 300 pN (ref. 7), the segment is removed from the filament where it belongs. As a result of each severing event, one filament is divided into two filaments.

5. Dynamic behaviors of ACPs.

ACP segments bind to binding sites located every 7 nm on actin segments at a constant rate, $k_{+,ACP}$ without preference for crosslinking angle. Unlike our previous studies[8], the unbinding of ACP segments from F-actin is not permitted to allow a network rupture to occur only via F-actin severing. If an actin segment disappears as a result of the F-actin severing, ACPs previously bound to the segment can bind to other F-actin.

Our assumption in the model that the unbinding event of ACPs from F-actin is not allowed may not look reasonable. However, this assumption can be justified as follows. It has been demonstrated *in vitro* that the unbinding force of single skeletal muscle α-actinin from F-actin ranged from 1.4 to 44 pN (18 pN on average) (ref. 9) or 40 to 80 pN (ref. 10), depending on the loading rate. On the other hand, the severing force of F-actin ranges between 200 pN and 600 pN, depending on the magnitude of twisting[7]. Assuming that 40 pN is the mean unbinding force of non-muscle α-actinin I from F-actin and that each α-actinin-F-actin bond is equally stressed, F-actin severing is expected to dominate the network rupture mechanism when there are more than 5-15 α-actinin molecules bound to a single filament. Using the mean length of F-actin in simulations (~4.2 μm in the 2D network and ~5.2 μm in the 3D network; **Fig. 6b,** caption) and the size of actin monomers (5.4 nm), the mean number of α-actinin molecules bound to a single actin filament is estimated to be more than 6 molecules under all tested conditions (minimum case: 6 molecules at R_X = 0.004; reference case: 124 molecules at R_X = 0.08). In addition, the mean F-actin length in the experiments is estimated to be ~4.6 μm (ref. 11) (the same actin concentration and buffer were used in this report), which is comparable

bioRxiv preprint doi: https://doi.org/10.1101/2025.05.18.654456; this version posted May 22, 2025. The copyright holder for this preprint (which was not certified by peer review) is the author/funder, who has granted bioRxiv a license to display the preprint in perpetuity. It is made available under aCC-BY 4.0 International license.

to the simulations. Therefore, it is reasonable to approximate that severing dominates the network rupture events in the simulations.

6. Dynamic behaviors of motors.

Motors are created by the self-assembly of backbone segments. Motor arms connected to the backbone points bind to binding sites on actin segments at a constant rate, $k_{+,\mathrm{M}} = 40N_\mathrm{h}$ s^{-1}. The walking ($k_{\mathrm{w,M}}$) and unbinding ($k_{\mathrm{u,M}}$) rates of the motor arms are determined rigorously by the parallel cluster model to capture the mechanochemical cycle of non-muscle myosin II[13,14]. The details of implementation and benchmarking of the parallel cluster model in our model were thoroughly described in our previous study[6]. $k_{\mathrm{w,M}}$ and $k_{\mathrm{u,M}}$ in the model are lower with a higher applied load, based on an assumption that motors behave as a catch bond. The unloaded walking velocity and stall force of motor arms are set to ~140 nm/s and ~5.7 pN, respectively. If an actin segment where motor arms are bound disappears due to the F-actin severing, motor arms can bind to other actin segments.

7. Interactions between the cytoskeletal elements and the membrane.

Repulsive forces are applied between all cytoskeletal elements and the membrane mesh to keep the cytoskeletal elements within the membrane. The repulsive forces originate from the potential shown in **Eq. S10** and the same strength, $\kappa_{\mathrm{r,MB}}$. Here, $r_{0,\mathrm{r}}$ is equal to the average of membrane thickness and the diameter of a neighboring cytoskeletal element, $r_{0,\mathrm{r}} = (d_{\mathrm{MB}} + d_i)/2$, where i is either A (actin), ACP, or M (motor).

A fraction of ACPs are selected at the beginning of simulations based on the strength of network-membrane coupling. The selected ACPs can feel an attractive force from the membrane if a distance between the ACPs and the membrane is smaller than $d_{\mathrm{ACP}} + d_{\mathrm{MB}}$. The attractive force is defined by the following harmonic potential:

$$U_{\mathrm{a,MB}} = \begin{cases} \frac{1}{2}\kappa_{\mathrm{a,MB}}\left(r_{12} - \frac{r_{0,\mathrm{a}}}{2}\right)^2 & \text{if } \frac{r_{0,\mathrm{a}}}{2} \le r_{12} < r_{0,\mathrm{a}} \\ 0 & \text{if } r < \frac{r_{0,\mathrm{a}}}{2} \text{ or } r \ge r_{0,\mathrm{a}} \end{cases} \tag{S11}$$

where $\kappa_{\mathrm{a,MB}}$ is the strength of the attractive force, and $r_{0,\mathrm{a}}$ is equal to $d_{\mathrm{ACP}} + d_{\mathrm{MB}}$**.** Note that the attractive force is always applied in a direction normal to the closest triangular mesh element, meaning that ACPs can freely slide along the membrane surface with the maintenance of the equilibrium distance from the membrane. Drag forces acting between ACPs and the membrane

bioRxiv preprint doi: https://doi.org/10.1101/2025.05.18.654456; this version posted May 22, 2025. The copyright holder for this preprint (which was not certified by peer review) is the author/funder, who has granted bioRxiv a license to display the preprint in perpetuity. It is made available under aCC-BY 4.0 International license.

are ignored for simplicity, meaning that ACPs bound to the membrane still experience a drag force defined by **Eq. S3**.

In the simulations, it is assumed that the membrane-bound ACPs dissociate from the membrane if a distance between them (r_{12}) becomes greater than $r_{0,\mathrm{a}}$. At this threshold distance, ACPs experience ~30 pN (**Eq. S11** and **Table S1**). In the experiments, α-actinin binds to the membrane through the interaction between the His-tag and Ni-NTA on the lipid. It has been measured by AFM that a dissociation force for His-tag-Ni-NTA interaction is >100 pN (ref. 12), which is larger than the unbinding force of α-actinin from F-actin (1.4-80 pN) (refs. 9,10). Therefore, it is more likely that the detachment of the cortex from the membrane is induced by the unbinding of α-actinin from F-actin rather than by the dissociation of α-actinin from the membrane. However, our assumption in the simulations is expected to have a minor effect on overall results because the dissociation force of ACP from the membrane in the model (~30 pN) is comparable to that of α-actinin from F-actin. This assumption helps reduce the computational cost.

8. Simulation setup.

At the beginning of each simulation, an actin network is created via the self-assembly processes of cytoskeletal elements. Actin concentration in all simulations is fixed at C_{A} = 10 μM, which is calculated using the entire volume within the membrane. The molar ratios of ACPs ($R_{\mathrm{X}} = C_{\mathrm{X}} / C_{\mathrm{A}}$) and motors ($R_{\mathrm{M}} = C_{\mathrm{M}}/C_{\mathrm{A}}$) specified in each simulation determine their amounts. The network is created as either a 2D network right beneath the membrane (i.e., within a space defined by a radial distance from the membrane center between 0.9 × r_{MB} and r_{MB} where r_{MB} is a membrane radius) or a 3D network in an entire space within the membrane (**Fig. 6b**). Note that only 27.1% of the space within the membrane is used for network assembly in case of the 2D network. During the network assembly, the membrane is frozen without a change in its spherical shape, and the network and membrane are coupled via attractive forces acting on ACPs selected at the beginning of simulations. After the network assembly, motors start walking to generate mechanical forces, and the membrane also starts deforming by thermal fluctuation and motor-generated forces.

9. Measurements of the formation and size of blebs.

To detect bleb formation, the curvature of the membrane is measured every 5 s. To calculate the curvature, we use the discrete mean curvature approximation with consideration of the

bioRxiv preprint doi: https://doi.org/10.1101/2025.05.18.654456; this version posted May 22, 2025. The copyright holder for this preprint (which was not certified by peer review) is the author/funder, who has granted bioRxiv a license to display the preprint in perpetuity. It is made available under aCC-BY 4.0 International license.

Voronoi area and common triangular edge length, as explained in detail in a previous study[15] (**Fig. S6a**). After calculating the curvature, the thresholding technique is used to detect blebs appropriately. To detect blebs accurately and exclude random picks, the threshold curvature was carefully chosen. A region with a high positive curvature surrounded by negative-curvature regions is considered a possible location for bleb formation. Then, distances between the apex of the identified bleb and the edges of the bleb where the curvature flips from positive to negative are measured, and then the average of the distances is calculated as the bleb size. Our algorithm can keep track of more than one bleb emerging in a simulation (**Fig. S6b-d**).

10. Measurement of tension acting on the actin network and the membrane.

To assess global tension acting on the actin network or the membrane, we consider *xy*, *yz*, and *zx* cross-sections that include the instantaneous centroid of the membrane for membrane tension or include the centroid of the network for network tension (**Fig. S6e**). All chains of the membrane mesh (for membrane tension) or all segments of F-actin, ACPs, and motors (for network tension) crossing each cross-section are selected. The magnitude of extensional forces acting on selected chains or segments, $F_{s,i}$, is calculated, which is either positive (= tensile) or negative (= compressive) (**Fig. S6f**). Then, an acute angle between the orientation of chains or segments and a direction normal to the cross-section, ϕ_i, is calculated. The sum of $F_{s,i} \cos\phi_i$ over all chains or segments is considered total tension acting on one cross-section. Then, it is normalized by the contour length of intersection between the network and the cross-section or by the contour length of intersection between the membrane and the cross-section. Global network/membrane tension is calculated by averaging the normalized tension measured on three cross-sections.

To evaluate time evolution of local tension developed near the identified bleb, we first find a time point when the bleb radius reaches 10% of the membrane radius. Then, all F-actin located beneath the bleb and within ~300 nm from the edges of the bleb is selected. The sum of extensional forces acting on these F-actin is calculated from the end of the network assembly (when motors start walking) till the time point when F-actin is selected.

11. Assessment of local areal density of membrane-bound ACPs.

First, the surface area of a spherical membrane is divided into quadrilateral and triangular surface elements using the spherical coordinate (**Fig. S6g**). Membrane nodes are allocated to the surface elements based on their positions. Since the distance between neighboring

bioRxiv preprint doi: https://doi.org/10.1101/2025.05.18.654456; this version posted May 22, 2025. The copyright holder for this preprint (which was not certified by peer review) is the author/funder, who has granted bioRxiv a license to display the preprint in perpetuity. It is made available under aCC-BY 4.0 International license.

membrane nodes is typically smaller than the size of one surface element, several membrane nodes are assigned to a single surface element. Then, the local areal density of membrane-bound ACPs on each surface element is calculated by dividing the total number of ACPs coupled to all the membrane nodes allocated to the surface element by the sum of the areas that the membrane nodes occupy. The area occupied by each membrane node is calculated by averaging the instantaneous area of three triangular membrane elements connected to the membrane node.

bioRxiv preprint doi: https://doi.org/10.1101/2025.05.18.654456; this version posted May 22, 2025. The copyright holder for this preprint (which was not certified by peer review) is the author/funder, who has granted bioRxiv a license to display the preprint in perpetuity. It is made available under aCC-BY 4.0 International license.

Table S1. List of parameters employed in the model.

Symbol	Definition	Value
$l_{0,\mathrm{A}}$	Length of an actin segment	1.4×10^{-7} [m]
d_{A}	Diameter of an actin segment	7.0×10^{-9} [m] (ref. 16)
$\theta_{0,\mathrm{A}}$	Bending angle formed by adjacent actin segments	0 [rad]
$\kappa_{\mathrm{s,A}}$	Extensional stiffness of F-actin	1.69×10^{-2} [N/m]
$\kappa_{\mathrm{b,A}}$	Bending stiffness of F-actin	2.64×10^{-19} [N·m] (ref. 2)
$\kappa_{\mathrm{r,A}}$	Strength of repulsive force between neighboring F-actin	1.69×10^{-3} [N/m]
$k_{\mathrm{n,A}}$	Nucleation rate of actin	1.0×10^{-6} [μM^{-1}s^{-1}]
$k_{+,\mathrm{A}}$	Polymerization rate of actin at the barbed end	60 [μM^{-1}s^{-1}]
F_{sev}	A minimum force for F-actin severing	300 [pN]
$l_{0,\mathrm{ACP}}$	Length of an ACP segment	2×10^{-8} [m] (ref. 17)
d_{ACP}	Diameter of an ACP segment	1.0×10^{-8} [m]
$\theta_{0,\mathrm{ACP}}$	Bending angle formed by two ACP segments	0 [rad]
$\kappa_{\mathrm{s,ACP}}$	Extensional stiffness of ACP	2.0×10^{-3} [N/m]
$\kappa_{\mathrm{b,ACP}}$	Bending stiffness of ACP	1.04×10^{-19} [N·m]
$k_{+,\mathrm{ACP}}$	Binding rate of an ACP segment	100 [s^{-1}]
$l_{0,\mathrm{M1}}$	Length of a motor backbone segment	4.2×10^{-8} [m]
d_{M1}	Diameter of a motor backbone segment	1.0×10^{-8} [m]
$\theta_{0,\mathrm{M}}$	Bending angle formed by motor backbone segments	0 [rad]
$\kappa_{\mathrm{s,M1}}$	Extensional stiffness of a motor backbone	1.69×10^{-2} [N/m]
$\kappa_{\mathrm{b,M}}$	Bending stiffness of a motor backbone	5.07×10^{-18} [N·m]
$l_{0,\mathrm{M2}}$	Equilibrium length 1 of a motor arm	1.0×10^{-8} [m]
$l_{0,\mathrm{M3}}$	Equilibrium length 2 of a motor arm	0 [m]
d_{M2}	Diameter of a motor arm	1.0×10^{-8} [m]
$\kappa_{\mathrm{s,M2}}$	Extensional stiffness 1 of a motor arm	1.0×10^{-3} [N/m]
$\kappa_{\mathrm{s,M3}}$	Extensional stiffness 2 of a motor arm	1.0×10^{-3} [N/m]
N_{h}	Number of heads represented by a motor arm	4
N_{arm}	Number of arms in a single motor	16
$k_{+,\mathrm{M}}$	Binding rate of a motor arm	$40N_{\mathrm{h}}$ [s^{-1}]
$l_{0,\mathrm{MB}}$	Length of a membrane mesh chain	7.0×10^{-8} [m]
d_{MB}	Thickness of a membrane mesh	5.0×10^{-8} [m]
$\theta_{0,\mathrm{MB}}$	Dihedral angle formed by two adjacent mesh elements	0 [rad]
$\kappa_{\mathrm{s,MB}}$	Extensional stiffness of a membrane mesh chain	1.0×10^{-4} [N/m]
$\kappa_{\mathrm{b,MB}}$	Bending stiffness of a membrane mesh	2.4×10^{-19} [N·m]
$A_{0,\mathrm{MB}}$	Area of one triangular mesh	2.12×10^{-15} [m^2]
$\kappa_{\mathrm{A,MB}}$	Strength of areal conservation	1.0×10^{-3} [N/m]
$V_{0,\mathrm{MB}}$	Volume within a membrane mesh	2.68×10^{-16} [m^3]
$\kappa_{\mathrm{V,MB}}$	Strength of volume conservation	1.0×10^{3} [N/m^2]
$\kappa_{\mathrm{a,MB}}$	Strength of an attractive force between a mesh and ACPs	1.0×10^{-3} [N/m]
$\kappa_{\mathrm{r,MB}}$	Strength of a repulsive force acting between all cytoskeletal elements and a mesh and between membrane mesh elements	5.1×10^{-3} [N/m]
Δt	Time step	1.15×10^{-4} [s]
μ	Viscosity of a medium	8.6 [Pa·s]
$k_{\mathrm{B}}T$	Thermal energy	4.142×10^{-21} [J]

bioRxiv preprint doi: https://doi.org/10.1101/2025.05.18.654456; this version posted May 22, 2025. The copyright holder for this preprint (which was not certified by peer review) is the author/funder, who has granted bioRxiv a license to display the preprint in perpetuity. It is made available under aCC-BY 4.0 International license.

Table S2. List of parameter values used for adopting "parallel cluster model" Note that we used slightly different values for $F_{0,\mathrm{M}}$, s, and k_m from those in the literatures (refs. 13, 14).

Symbol	Definition	Value
$k_{01,\mathrm{M}}$	A rate from unbound to weakly bound state	40 [s^{-1}]
$k_{10,\mathrm{M}}$	A rate from weakly bound to unbound state	2 [s^{-1}]
$k_{12,\mathrm{M}}$	A rate from weakly bound to post-power-stroke state	1000 [s^{-1}]
$k_{21,\mathrm{M}}$	A rate from post-power-stroke to weakly bound state	1000 [s^{-1}]
$k_{20,\mathrm{M}}$	A rate from post-power-stroke to unbound state	20 [s^{-1}]
$F_{0,\mathrm{M}}$	Constant for force dependence	5.04×10^{-12} [N]
E_{pp}	Free energy bias toward the post-power-stroke state	-60×10^{-21} [J]
E_{ext}	External energy contribution	0 [J]
s	Step size	7×10^{-9} [m]
k_m	Spring constant of the neck linkers	1.0×10^{-3} [N/m] ($=\kappa_{s,M3}$)

bioRxiv preprint doi: https://doi.org/10.1101/2025.05.18.654456; this version posted May 22, 2025. The copyright holder for this preprint (which was not certified by peer review) is the author/funder, who has granted bioRxiv a license to display the preprint in perpetuity. It is made available under aCC-BY 4.0 International license.

REFERENCES

1. Underhill P. T. & Doyle P. S. On the coarse-graining of polymers into bead-spring chains. *J. Nonnewton Fluid Mech.* **122,** 3–31 (2004).

2. Isambert H. *et al.* Flexibility of actin filaments derived from thermal fluctuations. *J. Biol. Chem.* **270,** 11437–11444 (1995).

3. Le S. *et al.* Mechanotransmission and mechanosensing of human alpha-actinin 1. *Cell Rep.* **21,** 2714–2723 (2017).

4. Tyska M. J. *et al.* Two heads of myosin are better than one for generating force and motion. *Proc. Natl Acad. Sci. USA* **96,** 4402–4407 (1999).

5. Sinard, J. H. Stafford, W. F. & Pollard, T. D. The mechanism of assembly of Acanthamoeba myosin-II minifilaments: minifilaments assemble by three successive dimerization steps. *J. Cell Biol.* **109,** 1537–1547 (1989).

6. Kim. T. Determinants of contractile forces generated in disorganized actomyosin bundles. *Biomech. Model Mechanobiol.* **14,** 345–355 (2015).

7. Tsuda, Y., Yasutake, H., Ishijima, A. & Yanagida, T. Torsional rigidity of single actin filaments and actin–actin bond breaking force under torsion measured directly by in vitro micromanipulation. *Proc. Natl Acad. Sci. USA* **93,** 12937–12942 (1996).

8. Jung, W., Murrell, M. P. & Kim, T. F-actin cross-linking enhances the stability of force generation in disordered actomyosin networks. *Comput. Part. Mech.* **2,** 317–327 (2015).

9. Miyata, H., Yasuda, R. & Kinosita, K. Strength and lifetime of the bond between actin and skeletal muscle α-actinin studied with an optical trapping technique. *Biochim. Biophys. Acta* **1290,** 83–88 (2003).

10. Ferrer, J. M., Lee, H., Chen, J., Pelz, B., Nakamura, F., Kamm, R. D. & Lang, M. J. Measuring molecular rupture forces between single actin filaments and actin-binding proteins. *Proc. Natl Acad. Sci. USA* **105,** 9221–9226 (2008).

11. Miyazaki, M., Chiba, M., Eguchi, H., Ohki, T. & Ishiwata, S. Cell-sized spherical confinement induces the spontaneous formation of contractile actomyosin rings *in vitro*. *Nat. Cell Biol.* **17,** 480–489 (2015).

bioRxiv preprint doi: https://doi.org/10.1101/2025.05.18.654456; this version posted May 22, 2025. The copyright holder for this preprint (which was not certified by peer review) is the author/funder, who has granted bioRxiv a license to display the preprint in perpetuity. It is made available under aCC-BY 4.0 International license.

12. Verbelen, C., Gruber, H. J. & Dufrêne, Y. F. The NTA–His6 bond is strong enough for AFM single-molecular recognition studies. *J. Mol. Recognit.* **20,** 490–494 (2007).

13. Erdmann, T. Albert, P. J. & Schwarz, U. S. Stochastic dynamics of small ensembles of non-processive molecular motors: The parallel cluster model. *J. Chem. Phys.* **139,** 175104 (2013).

14. Erdmann, T. & Schwarz, U. S. Stochastic force generation by small ensembles of myosin II motors. *Phys. Rev. Lett.* **108,** 188101 (2012).

15. Meyer, M., Desbrun, M., Schröder, P. & Barr, A. H. *in Visualization and Mathematics III* (eds Hege, H.-C. & Polthier, K.) 35–57 (Springer, 2003).

16. Kishino, A. & Yanagida, T. Force measurements by micromanipulation of a single actin filament by glass needles. *Nature* **334,** 74–76 (1988).

17. Meyer, R. K. & Aebi, U. Bundling of actin filaments by alpha-actinin depends on its molecular length. *J. Cell Biol.* **110,** 2013–2024 (1990).

bioRxiv preprint doi: https://doi.org/10.1101/2025.05.18.654456; this version posted May 22, 2025. The copyright holder for this preprint (which was not certified by peer review) is the author/funder, who has granted bioRxiv a license to display the preprint in perpetuity. It is made available under aCC-BY 4.0 International license.

MOVIE CAPTIONS

Movie S1. Liposome showing bleb formation induced by the contraction of a volume-spanning actin network (3D network). The liposome contains 10 μM actin, 2 μM His-α-actinin, 1 μM SMM (10% Alexa488-labeled), and 1.4 × 10^{-4} unit ml^{-1} ZIPK, but did not contain methylcellulose. The images were taken with a confocal microscope. Scale bar, 10 μm.

Movie S2. Liposome showing local actin network contraction but no detectable deformation (2D network). The liposome contains 10 μM actin, 1 μM SMM, 1.4 × 10^{-4} unit ml^{-1} ZIPK, and methylcellulose, but did not contain His-α-actinin. The images were taken with an epi-fluorescence microscope. Scale bar, 10 μm.

Movie S3. Liposome showing weak deformation (2D network). The liposome contains 10 μM actin, 0.5 μM His-α-actinin, 1 μM SMM, 1.4 × 10^{-4} unit ml^{-1} ZIPK, and methylcellulose. The images were taken with an epi-fluorescence microscope. Scale bar, 10 μm.

Movie S4. Liposome forming a single bleb (2D network). The liposome contains 10 μM actin, 5 μM His-α-actinin, 1 μM SMM, 1.4 × 10^{-4} unit ml^{-1} ZIPK, and methylcellulose. The images were taken with an epi-fluorescence microscope. Scale bar, 10 μm.

Movie S5. Liposome forming two blebs (2D network). The liposome contains 10 μM actin, 1 μM His-α-actinin, 1 μM SMM, 1.4 × 10^{-4} unit ml^{-1} ZIPK, and methylcellulose. The images were taken with an epi-fluorescence microscope. Scale bar, 10 μm.

Movie S6. Liposome showing the contraction of an actin network without membrane deformation (2D network). The liposome contains 10 μM actin, 2 μM α-actinin, 1 μM SMM, 1.4 × 10^{-4} unit ml^{-1} ZIPK, and methylcellulose. The images were taken with an epi-fluorescence microscope. Scale bar, 10 μm.

Movie S7. Liposome forming a single bleb initiated by the rupture of the cortical actin network

bioRxiv preprint doi: https://doi.org/10.1101/2025.05.18.654456; this version posted May 22, 2025. The copyright holder for this preprint (which was not certified by peer review) is the author/funder, who has granted bioRxiv a license to display the preprint in perpetuity. It is made available under aCC-BY 4.0 International license.

(2D network). The liposome contains 10 µM actin, 2 µM His-α-actinin, 1 µM SMM, 1.4 × 10^{-4} unit ml^{-1} ZIPK, and methylcellulose. The images were taken with an epi-fluorescence microscope. Scale bar, 10 µm.

Movie S8. Liposome forming a single bleb initiated by the detachment of the cortical actin network from the membrane (2D network). The liposome contains 10 µM actin, 2 µM His-α-actinin, 1 µM SMM, 1.4 × 10^{-4} unit ml^{-1} ZIPK, and methylcellulose. The detached cortical actin network completed the contraction without any detectable rupture. The images were taken with an epi-fluorescence microscope. Scale bar, 10 µm.

Movie S9. Liposome forming a single bleb initiated by the detachment of the cortical actin network from the membrane, followed by spontaneous rupture of the contracting actin network (2D network). The liposome contains 10 µM actin, 2 µM His-α-actinin, 1 µM SMM, 1.4 × 10^{-4} unit ml^{-1} ZIPK, and methylcellulose. After the detachment of the cortical actin network, the network was spontaneously ruptured (t = 31 min). The images were taken with an epi-fluorescence microscope. Scale bar, 10 µm.

Movie S10. Liposome forming a single bleb with a lower concentration of ZIPK (2D network). The liposome contains 10 µM actin, 2 µM His-α-actinin, 1 µM SMM, 0.53 × 10^{-4} unit ml^{-1} ZIPK, and methylcellulose. The images were taken with an epi-fluorescence microscope. Scale bar, 10 µm.

Movie S11. Liposome showing neither detectable deformation nor actin network contraction (3D network). The liposome contains 10 µM actin, 1 µM SMM, and 1.4 × 10^{-4} unit ml^{-1} ZIPK, but did not contain His-α-actinin. The images were taken with an epi-fluorescence microscope. Scale bar, 10 µm.

Movie S12. Liposome showing weak deformation (3D network). The liposome contains 10 µM actin, 0.5 µM His-α-actinin, 1 µM SMM, and 1.4 × 10^{-4} unit ml^{-1} ZIPK. The images were taken with an epi-fluorescence microscope. Scale bar, 10 µm.

bioRxiv preprint doi: https://doi.org/10.1101/2025.05.18.654456; this version posted May 22, 2025. The copyright holder for this preprint (which was not certified by peer review) is the author/funder, who has granted bioRxiv a license to display the preprint in perpetuity. It is made available under aCC-BY 4.0 International license.

Movie S13. Liposome forming multiple blebs (3D network). The liposome contains 10 µM actin, 2 µM His-α-actinin, 1 µM SMM, and 1.4 × 10^{-4} unit ml^{-1} ZIPK. The images were taken with an epi-fluorescence microscope. Scale bar, 10 µm.

Movie S14. Liposome forming a single bleb (3D network). The liposome contains 10 µM actin, 3 µM His-α-actinin, 1 µM SMM, and 1.4 × 10^{-4} unit ml^{-1} ZIPK. The images were taken with an epi-fluorescence microscope. Scale bar, 10 µm.

Movie S15. Bleb formation in a larger system with 16 µm in diameter (2D network). Motor density (R_M) is 0.01, network connectivity (R_X) is 0.01, and actin-membrane coupling strength (R_C) is 0.01.

Movie S16. Formation of a single bleb under the reference condition (2D network). Motor density (R_M) is 0.01, network connectivity (R_X) is 0.08, and actin-membrane coupling strength (R_C) is 0.08. Bleb formation was initiated by the rupture mechanism.

Movie S17. Successive severing events of F-actin driving the expansion of a rupture formed in the actin network (2D network). On the left, local forces acting on the network are visualized via color scaling. On the right, F-actin, actin cross-linking protein, and motor are visualized using different colors.

Movie S18. No deformation in the absence of actin-membrane coupling (2D network). Motor density (R_M) is 0.01, network connectivity (R_X) is 0.08, and actin-membrane coupling strength (R_C) is 0. The network contracted into a smaller cluster without noticeable membrane deformation. Local forces are visualized via color scaling.

Movie S19. Detachment-induced formation of a single bleb with low actin-membrane coupling strength (2D network). Motor density (R_M) is 0.01, network connectivity (R_X) is 0.08, and actin-membrane coupling strength (R_C) is 0.016. Local forces are visualized via color scaling.

bioRxiv preprint doi: https://doi.org/10.1101/2025.05.18.654456; this version posted May 22, 2025. The copyright holder for this preprint (which was not certified by peer review) is the author/funder, who has granted bioRxiv a license to display the preprint in perpetuity. It is made available under aCC-BY 4.0 International license.

Movie S20. Rupture-induced formation of a single bleb with high actin-membrane coupling strength (2D network). Motor density (R_M) is 0.01, network connectivity (R_X) is 0.08, and actin-membrane coupling strength (R_C) is 0.048. Local forces are visualized via color scaling.

Movie S21. Weak deformation with low network connectivity (2D network). Motor density (R_M) is 0.01, network connectivity (R_X) is 0.006, and actin-membrane coupling strength (R_C) is 0.006. Network contraction was observed, but the membrane was not deformed noticeably. Local forces are visualized via color scaling.

Movie S22. Rupture-induced formation of a single bleb with intermediate network connectivity (2D network). Motor density (R_M) is 0.01, network connectivity (R_X) is 0.06, and actin-membrane coupling strength (R_C) is 0.06. The network underwent severe aggregation into a small cluster. Local forces are visualized via color scaling.

Movie S23. Weak deformation with high network connectivity (2D network). Motor density (R_M) is 0.01, network connectivity (R_X) is 0.4, and actin-membrane coupling strength (R_C) is 0.4. The membrane was deformed, but a bleb was not formed. Local forces are visualized via color scaling.

Movie S24. Formation of multiple blebs (3D network). Motor density (R_M) is 0.01, network connectivity (R_X) is 0.08, and actin-membrane coupling strength (R_C) is 0.08. Two blebs were formed by the rupture mechanism. Local forces are visualized via color scaling.

Movie S25. Distribution, orientation, and force of motors in a 2D network. Motor density (R_M) is 0.01, network connectivity (R_X) is 0.08, and actin-membrane coupling strength (R_C) is 0.08.

Movie S26. Distribution, orientation, and force of motors in a 3D network. Motor density (R_M) is 0.01, network connectivity (R_X) is 0.08, and actin-membrane coupling strength (R_C) is 0.08.
